# The economic burden of households affected by tuberculosis in Brazil: First national survey results, 2019-2021

**DOI:** 10.1371/journal.pone.0287961

**Published:** 2023-12-13

**Authors:** Ethel Leonor Noia Maciel, Letícya dos Santos Almeida Negri, Leticia Molino Guidoni, Geisa Carlesso Fregona, Fernanda Dockhorn Costa Johansen, Mauro Niskier Sanchez, Adriana da Silva Rezende Moreira, Fredi Alexander Diaz-Quijano, Maiko Tonini, Eliana Zandonade, Julia Ershova, Peter Nguhiu, Inés Garcia Baena

**Affiliations:** 1 Laboratory of Epidemiology Federal University of Espírito Santo, UFES, Vitória, ES, Brazil; 2 Federal University of Espírito Santo, UFES, Vitória, ES, Brazil; 3 Cassiano Antônio Moraes University Hospital – HUCAM, Vitória, ES, Brazil; 4 National TB Programme, Ministry of Health, Brasília, Brazil; 5 University of Brasília, UNB, Brasília, DF, Brazil; 6 Federal University of Rio de Janeiro, UFRJ, Rio de Janeiro, Brazil; 7 University of São Paulo, USP, São Paulo, Brazil; 8 Federal University of Espírito Santo, ES, Brazil; 9 Centers for Disease Control and Prevention, Atlanta, Georgia, United States of America; 10 KEMRI- Wellcome Trust Research Program, Health Economics Research Unit, Nairobi, Kenya; 11 World Health Organization, Global TB Programme, Geneva, Switzerland; Universidade Federal do Rio Grande do Norte, BRAZIL

## Abstract

**Background:**

One of the three main targets of the World Health Organization (WHO) End TB Strategy (2015-2035) is that no tuberculosis (TB) patients or their households face catastrophic costs (defined as exceeding 20% of the annual household income) because of the disease. Our study seeks to determine, as a baseline, the magnitude and main drivers of the costs associated with TB disease for patients and their households and to monitor the proportion of households experiencing catastrophic costs in Brazil.

**Methods:**

A national cross-sectional cluster-based survey was conducted in Brazil in 2019-2021 following WHO methodology. TB patients of all ages and types of TB were eligible for the survey. Adult TB patients and guardians of minors (<18 years old) were interviewed once about costs, time loss, coping measures, income, household expenses, and asset ownership. Total costs, including indirect costs measured as reported household income change, were expressed as a percentage of annual household income. We used descriptive statistics to analyze the cost drivers and multivariate logistic regression to determine factors associated with catastrophic costs.

**Results:**

We interviewed 603 patients, including 538 (89%) with drug-sensitive (DS) and 65 (11%) with drug-resistant (DR) TB. Of 603 affected households, 48.1% (95%CI: 43–53.2) experienced costs above 20% of their annual household income during their TB episode. The proportion was 44.4% and 78.5% among patients with DS- and DR-TB, respectively. On average, patients incurred costs of US$1573 (95%CI: 1361.8–1785.0) per TB episode, including pre-diagnosis and post-diagnosis expenses. Key cost drivers were post-diagnosis nutritional supplements (US$317.6, 95%CI: 232.7–402.6) followed by medical costs (US$85.5, 95%CI: 54.3–116.5) and costs of travel for clinic visits during treatment (US$79.2, 95%CI: 61.9–96.5). In multivariate analysis, predictors of catastrophic costs included positive HIV status (aOR = 3.0, 95%CI:1.1–8.6) and self-employment (aOR = 2.7, 95%CI:1.1–6.5); high education was a protective factor (aOR = 0.1, 95%CI:0.0–0.9).

**Conclusions:**

Although the services offered to patients with TB are free of charge in the Brazilian public health sector, the availability of free diagnosis and treatment services does not alleviate patients’ financial burden related to accessing TB care. The study allowed us to identify the costs incurred by patients and suggest actions to mitigate their suffering. In addition, this study established a baseline for monitoring catastrophic costs and fostering a national policy to reduce the costs to patients for TB care in Brazil.

## Introduction

Estimating the costs borne by TB-affected households and monitoring progress towards the World Health Organization’s (WHO) End TB Strategy targets are essential for Brazil. Brazil achieved the targets of the Millennium Development Goals (MDG) for TB control before the 2015 deadline, but the WHO End TB Strategy and Sustainable Development (SDG) goals are more ambitious and are unlikely to be achieved without significant and profound changes in healthcare and social protection policies, nor without changes in the regional economic and political context [1, 2].

In 2015, WHO member states adopted the End TB strategy; one of its three goals was to move towards zero TB-affected households suffering catastrophic costs by 2035 [3–5]. By September 2021, WHO reported that 25 countries had completed a national survey to measure costs borne by TB-affected households and the proportion of households incurring costs due to TB that were above 20% of annual household income/expenditure, the indicator defined in the End TB Strategy [1, 6, 7]. The pooled weighted percentage of TB-affected households that experienced TB related costs above 20% of household income/expenditure was 47.3% (95% CI: 33.3-61.3%) while country estimates ranged from 13% (95% confidence interval [CI]: 10–17%) in El Salvador to 92% (95% CI: 86–97%) in Solomon Islands [6]. These results demonstrate that the economic burden faced by TB-affected households is influenced by the country’s progress towards universal health care [8–24]. It has been well documented too that TB disproportionately impacts the poorest and most vulnerable in the population, who experience higher proportionate costs and greater impact on their vulnerability [1–6].

Brazil has a population of 213 million people; 4.6% of the population were living on less than US$1.90 a day and 19,6% on less than PPP$5.50 a day (the poverty threshold for Upper-middle -income countries in 2019 [25, 26]. The incidence of TB in Brazil was 45 (95% CI: 38-52) per 100,000 population in 2020 and 46 (95%CI: 39-53) per 100,000 population in 2019 [6]. In 2020, fewer TB cases (82,930) were notified compared to 2019 (85,154) given COVID-19-related TB service disruptions that caused unprecedented health challenges [2, 4].

In 2018, 10% of the gross domestic product (GDP) was invested in health in Brazil, but the GDP growth has been only 2% since 2015; with the COVID-19 pandemic and compromised GDP growth, the prioritization of government health spending is of particular importance [27]. In 2018, Brazil spent US$848 per capita on health, of which US$234 (27.6%) was financed out-of-pocket [28]. Brazil, with the Universal Health Coverage (UHC) service coverage index(SDG 3.8.1) of 79 ranks high globally but with 26% of population facing catastrophic health expenditures (SDG 3.8.2) it is worse off than the global average (11.8% in 2017) [29].

TB patients in Brazil have access to free TB diagnosis and treatment services through the Unified Health System (SUS), provided the patient is notified to official institutions. In 2019, TB care was delivered to 92,500 patients across 13,876 health centres, including 11,004 Primary Health Services (PHS) and 2,872 Specialised Health Service (SHS) facilities. Based on country reported TB program expenditures, the Global TB Report 2021 estimated that delivering TB care in Brazil in 2020 cost US$509 and US$3,913 per patient for drug-susceptible TB (DS-TB) and drug-resistant TB (DR-TB) respectively [6]. However, even with this framework of services, patients may incur out-of-pocket expenses while in care and income loss, compromising treatment access and adherence.

In Brazil, given the level of UHC, understanding the level of medical and non-medical expenditures, and indirect costs incurred by TB patients is necessary. A study by Rudgard W.E. et al performed in Rio de Janeiro amongst drug-resistant TB patients found that lost income was the key driver of economic burden during the TB episode, with a loss of US$6,207(sd = 6,671) per household, representing 81% (sd = 54%) of annual household income [23]. A study by Guidoni L.M. et al, conducted in five large cities in Brazil, found that on average income of participants declined by 11% during the TB episode and 41% faced direct and indirect costs that were higher than 20% of self-reported household income prior to TB [24].

Our study is the first national TB patient cost survey implemented in Brazil. It was conducted following WHO methodology and aimed to document the magnitude and main drivers of the costs incurred by TB patients and their households, and to establish the baseline proportion of TB-affected households that incur catastrophic total costs due to TB [7]. The survey’s findings could be used as a basis for actions to reduce financial barriers for accessing care and to minimize the adverse socioeconomic impacts of TB in Brazil.

## Methods

### Study setting

Brazil is the fifth largest country in the world by area (8,515 million km^2^), and the largest in South America. Brazil is divided into five administrative regions (North, Northeast, South, Southeast and Central-West), 26 states, 27 federative units and 5,570 municipalities. It ranks 84th in the world on the Human Development Index (HDI) scale, with 84.4% of the population living in urban areas [25–27].

Brazil has two public social protection policies that cover the entire population and are administered by independent systems. The Unified Health System (SUS) provides all health services free of charge, and the National Social Assistance System (SUAS) provides the guarantee of a minimum social protection for people living in poverty. The SUAS includes the Bolsa Familia Programme (BFP) - a cash transfer program for families in poverty, and Continuous Cash Benefit (BPC) program focused on the provision of sick pay, utility vouchers, etc. Around 70% of the population is covered by at least one social protection benefit. In response to the COVID-19 pandemic, an additional benefit (emergency assistance) was provided to support people in situations of social vulnerability. These financial support amounts ranged between US$20 (gas voucher) to US$213.2 per month from BPC [23, 30–32].

Tuberculosis is the world’s second top infectious killer after COVID-19, claiming close to 4100 lives a day). It is the leading killer of people with HIV and a major contributor of antimicrobial resistance related deaths [4]. In 2020 and 2021, the pandemic has had enormous consequences for health, society and the economy [6]. These include impacts on the provision of and access to essential TB services, TB notification, incidence and mortality as well as a reduced amount of funds allocated to TB care [6]. Operational research including our study also experienced disruptions. Due to logistical challenges during the COVID-19 pandemic, our study had two interruption periods: the first one was from March to August 2020, and the second one was from December 2020 to the end of March 2021.

### Study design, population, and sample size

We conducted a nationally representative health facility-based single-stage cluster-sampled survey, with retrospective data collection and cost extrapolation within the treatment period. The WHO provides a handbook with a standardized methodology for conducting health facility-based, cross-sectional surveys to assess the direct and indirect costs incurred by TB patients and their households [7]. The handbook outlines the study design, recommended sampling approaches, data collection and management, and recommended standard analyses. In line with this, the survey was designed to calculate the appropriate sample for a descriptive cluster survey that would estimate (with a power of 0.8) a proportional outcome measure (i.e catastrophic incidence headcount, estimated value of 50%) to an absolute precision of +/- 0.025. To account for the cluster design, we assumed a design effect of 2 and inflated the sample size by 5% to account for potential unit level non response, to arrive at a sample size of 760 TB patients recruited from 46 clusters (health facilities).

However, to ensure that unique characteristics and outcomes were captured in facilities that were sampled, the sampling frame was stratified into high enrolment and low enrolment strata. To do this, the annual notifications of tuberculosis patients in 2017 for each cluster were summed up within respective municipalities, and facilities in those municipalities with total notifications less than 37 per annum were classified as group 2 clusters while the rest were classified as group 1 clusters. Facilities in the group 2 stratum accounted for 22.9% of all notifications, but any cluster selected from this group would require longer time to recruit enough participants given the target of 20 participants per cluster. Therefore, the facilities (clusters) in group 2 stratum were oversampled with fewer participants enrolled in each cluster by design compared to group 1 stratum, to provide a greater chance of capturing between cluster variance in the survey.

In group 1, 36 clusters were sampled with probability proportional to cases notified from where 20 patients would be consecutively sampled: 12 patients from a Primary Health Care (PHC) facility and (where present) 8 from a Special Care (SC) facility. This stratification within group 1 was considered necessary given that the characteristics of people treated in PHC and in SC facilities were expected to vary. The ratio of patients sampled from each of these facility types was designed to match the national ratio of clinic attendance in 2017.

In group 2, ten clusters were selected. Since majority of these facilities had enrolled less than 3 patients (and the facility with the largest number enrolled had 8), sampling via probability proportion to size wouldn’t serve the purpose to allow diversity in cluster sampling, therefore these were sampled with constant probability. 4 patients were thereafter to be consecutively sampled from these facilities, noting that these facilities in group 2 were PHC facilities.

In summary, the sample selection was geared to represent the capacity (> = 35 or < 35 notified cases in 2017) as well as type of health care facility (PHC or SC). The under- and over-enrollment was adjusted at the time of analysis through sampling weights computed for each cluster from this sampling frame, and the whole process was embedded in a reproducible sampling script developed in R.

All adults and children currently on TB treatment for any type of TB that had completed at least 14 days of treatment, either in the intensive or continuation phase, were eligible to participate in the survey. Incarcerated persons, unaccompanied minors, and those on treatment for less than 14 days in the intensive or continuation phase were not enrolled in the study.

### Data collection

From September 2019 to April 2021 eligible patients from the selected clusters were invited to participate in the study. We used a standardized questionnaire developed by the WHO, adapted to the Brazilian context [7] (S1 File). Interviewers were trained during three training sessions at the Federal University of Espírito Santo (UFES) in Vitória, and travelled to collect data across the country. Data were collected on paper and subsequently entered, into the web-based data collection system [33].

Each eligible adult TB patient and guardian (legal companion) of eligible minor TB patient (<18 years old) was interviewed only once to obtain clinical and sociodemographic information, costs incurred due to TB disease (medical, non-medical and indirect), time loss, coping strategies and social support provided to mitigate costs related to TB treatment. Patients self-reported individual and household income (pre-disease, at diagnosis, and during treatment), annual household consumption, and household assets were collected following WHO definitions and the Instituto Brasileiro de Geografia e Estatística (IBGE) pre-tested assets questionnaire [34, 35]. Only patients interviewed during the intensive phase were questioned on pre-diagnostic costs and time loss, including during the diagnosis pathway. Costs, income, and expenses were collected in national currency (Real) and later converted using US$1 = 5.16 BRL according to the conversion rate in 2021 [36].

### Data analysis

The analysis followed the standard analysis plan outlined in the WHO Patient Cost Survey Handbook, standardising the computation of the diagnosis and treatment costs incurred by TB patients and their households per TB episode [7]. The result tables present the survey design adjusted estimates of mean costs with accompanying confidence intervals, which were obtained by using the survey package in R version 4.0.1 (R Foundation for Statistical Computing, Vienna, Austria) [37, 38]. However, results Tables 1 and 2 (the ones labelled as demographic and clinical survey participant characteristics) are presented in non-adjusted form since they represent the sample’s characteristics and are not to be interpreted as population representative characteristics.

**Table 1:** Socio-demographic characteristics of survey participants by TB drug-resistance status. Brazil national TB patient cost survey 2019-2021.

Category	TB patients (first-line treatment) (N = 538)	Patients with drug-resistant TB (N = 65)	Total (N = 603)
**Age category**			
**0-14**	15 (2.8%)	0 (0.0%)	15 (2.5%)
**15-24**	70 (13.0%)	5 (7.7%)	75 (12.4%)
**25-34**	93 (17.3%)	14 (21.5%)	107 (17.7%)
**35-44**	118 (21.9%)	19 (29.2%)	137 (22.7%)
**45-54**	109 (20.3%)	14 (21.5%)	123 (20.4%)
**55-64**	93 (17.3%)	6 (9.2%)	99 (16.4%)
**>65**	40 (7.4%)	7 (10.8%)	47 (7.8%)
**Sex**			
**Female**	208 (38.7%)	24 (36.9%)	232 (38.5%)
**Male**	330 (61.3%)	41 (63.1%)	371 (61.5%)
**Education level**			
**no schooling (up to 3 years)**	68 (12.6%)	17 (26.2%)	85 (14.1%)
**4 to 7 years**	147 (27.3%)	13 (20.0%)	160 (26.5%)
**8 to 10 years**	126 (23.4%)	19 (29.2%)	145 (24.0%)
**11 to 14 years**	148 (27.5%)	14 (21.5%)	162 (26.9%)
**Secondary or higher**	49 (9.1%)	2 (3.1%)	51 (8.5%)
**Employment status: Pre-disease^1^**			
**Employed (formal)**	194 (36.1%)	16 (24.6%)	210 (34.9%)
**Self employed**	170 (31.7%)	26 (40.0%)	196 (32.6%)
**Student/Retired/Others**	51 (9.5%)	5 (7.7%)	56 (9.3%)
**Unemployed**	122 (22.7%)	18 (27.7%)	140 (23.3%)
**Household size**			
**Mean (SD)**	3.5 (2.1)	3.2 (1.8)	3.4 (2.1)
**Median (IQR)**	3.0 (2.0, 4.0)	3.0 (2.0, 4.0)	3.0 (2.0, 4.0)
**Private health insurance**			
**No**	442 (82.2%)	61 (93.8%)	503 (83.4%)
**Yes**	96 (17.8%)	4 (6.2%)	100 (16.6%)
**Social protection assistance**			
**No**	337 (62.6%)	34 (52.3%)	371 (61.5%)
**Yes**	201 (37.4%)	31 (47.7%)	232 (38.5%)
**Interview time (COVID-19)^2^**			
**During COVID-19**	379 (70.4%)	44 (67.7%)	423 (70.1%)
**Before COVID-19**	159 (29.6%)	21 (32.3%)	180 (29.9%)
**Place of first Diagnosis^3^**			
**Basic Health Unit**	86 (16.0%)	13 (20.0%)	99 (16.4%)
**Basic Health Unit with TB program unit (PCT)**	5 (0.9%)	1 (1.5%)	6 (1.0%)
**Family Health Unit**	99 (18.4%)	15 (23.1%)	114 (18.9%)
**Family Health Unit with PCT**	7 (1.3%)	0 (0.0%)	7 (1.2%)
**Private Health Service**	68 (12.6%)	5 (7.7%)	73 (12.1%)
**Public hospital**	149 (27.7%)	20 (30.8%)	169 (28.0%)
**Tuberculosis Reference Unit**	71 (13.2%)	9 (13.8%)	80 (13.3%)
**Urgency and Emergency Unit**	40 (7.4%)	1 (1.5%)	41 (6.8%)
**Other**	13 (2.4%)	1 (1.5%)	14 (2.3%)
**Service type**			
**Primary Health Service (PHS)**	308 (57.2%)	17 (26.2%)	325 (53.9%)
**Specialized Health Service (SHS)**	230 (42.8%)	48 (73.8%)	278 (46.1%)

SD: standard deviation; PCT = TB Programme unit. PHS: primary health service. SHS: specialized health service.

^1^ Categories of employment were reclassified as follows: 1) Retired, student, homemaker = other +student; 2) Unemployed = unemployed worker receives help from household member + none; 3) Self-employment = self-employed; and 4) Formal employment= self-employed + employment in the public sector + employment in the private sector +army etc + working.

^2^ First case of COVID-19 was reported in February 2020.

^3^ PCT = TB Programme unit.

**Table 2:** Clinical characteristics of survey participants by TB drug-resistance status. Brazil national TB patient cost survey 2019-2021.

Category	TB patients (first-line treatment) (N = 538)	DR-TB patients (N = 65)	Total (N = 603)
**Type of diagnosis of TB**			
**Extra pulmonary TB**	89 (16.5%)	4 (6.2%)	93 (15.4%)
**Pulmonary + Extra pulmonary TB**	8 (1.5%)	2 (3.1%)	10 (1.7%)
**Pulmonary TB**	441 (82.0%)	59 (90.8%)	500 (82.9%)
**Treatment registration group**			
**New**	463 (86.1%)	37 (56.9%)	500 (82.9%)
**Previously treated**	23 (4.3%)	20 (30.8%)	43 (7.1%)
**Relapse**	52 (9.7%)	8 (12.3%)	60 (10.0%)
**HIV status**			
**Positive**	50 (9.3%)	10 (15.4%)	60 (10.0%)
**Negative**	430 (79.9%)	51 (78.5%)	481 (79.8%)
**Unknown**	58 (10.8%)	4 (6.2%)	62 (10.3%)
**Treatment phase**			
**Intensive phase**	207 (38.5%)	15 (23.1%)	222 (36.8%)
**Continuation phase**	331 (61.5%)	50 (76.9%)	381 (63.2%)
**Duration of intensive phase (month)**			
**Mean (Sd)**	2.1 (0.6)	4.6 (2.9)	2.3 (1.3)
**Median (IQR)**	2.0 (2.0, 2.0)	2.0 (2.0, 8.0)	2.0 (2.0, 2.0)
**Duration of continuation phase (month)**			
**Mean (SD)**	4.5 (1.4)	9.0 (3.6)	4.9 (2.3)
**Median (IQR)**	4.0 (4.0, 4.0)	10.0 (6.0, 10.0)	4.0 (4.0, 4.0)
**Modality of TB treatment**			
**Directly observed therapy (DOT)**	73 (13.6%)	8 (12.3%)	81 (13.4%)
**Self administered**	358 (66.5%)	45 (69.2%)	403 (66.8%)
**Self administered + DOT**	107 (19.9%)	12 (18.5%)	119 (19.7%)
**Hospitalization**			
**No**	463 (86.1%)	54 (83.1%)	517 (85.7%)
**Yes**	75 (13.9%)	11 (16.9%)	86 (14.3%)
**Previous TB episode**			
**No**	458 (85.1%)	35 (53.8%)	493 (81.8%)
**Yes**	80 (14.9%)	30 (46.2%)	110 (18.2%)
Comorbidities*			
**No**	392 (72.9%)	40 (61.5%)	432 (71.6%)
**Yes**	146 (27.1%)	25 (38.5%)	171 (28.4%)
**Time from onset of symptoms until diagnosis (in weeks)**			
**Mean (sd)**	9.3 (11.8)	22.6 (36.2)	9.8 (13.6)
**Median (IQR)**	4.0 (2.0, 12.0)	4.0 (3.5, 23.0)	4.0 (2.0, 12.0)

SD: standard deviation; IQR: Inter-quartile range; DR-TB: drug-resistant TB

Source

*Comorbidities include diabetes, hepatitis, and kidney disease.

### Costs borne by TB-affected households

Extrapolation of costs beyond a participant’s current treatment phase was done based on the median costs incurred by other patients and their households in the alternative treatment phase at the time of the interview. Total episode costs were estimated as the sum of direct medical (out-of-pocket payment for TB services, net of any reimbursements), direct non-medical (out-of-pocket payments made by TB-affected patient or guardian related to transportation, accommodation, food, nutritional supplements etc., net of any reimbursements) and indirect costs. We estimated indirect costs per TB episode using the output approach, based on the household’s income changes during the episode [7].

### Income and poverty level changes

Annualised monthly self-reported household income was used as the primary measure for determining household ability to pay. This is usual practice in surveys following WHO survey methods. In sensitivity analysis we also explored using household assets to estimate household’s ability to pay as an alternative method to measure the proportion of households experiencing catastrophic costs. We did not explore using household reported expenditure as an ability to pay because in this survey household expenditure data was more incomplete and unreliable [7].

We evaluated pre-disease household poverty levels by comparing daily income (calculated from self-reported household monthly income and comparing against the international poverty threshold of US$1.90 purchasing power parity adjusted dollars (converted to Brazilian Real (BRL) in 2011, then inflated using the ratio of consumer price index values in 2011 vs. 2020) as reported in the international finance statistics database [36].

To assess the possible impact of COVID-19 pandemic on the financial burden experiences by TB patients and their households, we compared information collected from patients interviewed before and during the pandemic.

### Catastrophic costs

**Fig 1:**
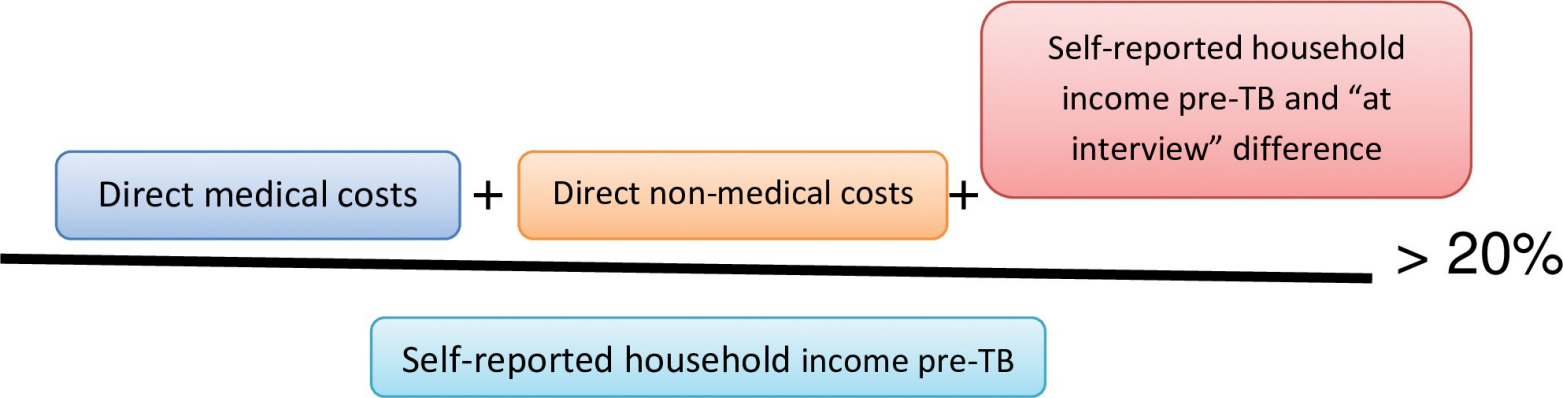
Estimation of catastrophic costs in tuberculosis.

We measured the proportion of households experiencing catastrophic costs due to TB among enrolled individuals. Catastrophic costs were defined as TB-related costs (direct and indirect) exceeding 20% of the annual household income, as shown in Fig 1.

Each TB-affected household was given a binary value to determine whether or not it incurred catastrophic costs according to this definition. Clustering effects associated with sampling methods were considered in estimating the overall proportion of catastrophic costs.

### Coping strategies, employment changes, and social consequences of TB episode

Each coping strategy was examined as a binary measure: whether or not the family used this strategy and the cost of the coping strategy. Similarly, binary measures of social impact and perceived impoverishment were used to estimate the proportion of households impacted. Employment status pre- and post-diagnosis was evaluated to estimate changes in unemployment levels amongst survey participants.

### Ethical approval

This study was approved by the Brazil National Research Ethics Council (CONEP) under Opinion n° 4.452641; by the World Health Organization through Opinions N° CGH HSR Tracking #:2019-167, PAHOERC-2019-0026, and by the U.S. Centers for Disease Control and Prevention (CDC), Center for Global Health (CGH) (Project number: 2018-277). The study was reviewed in accordance with the U.S. CDC human research protection procedures and determined to be research, but CDC investigators did not interact with human subjects nor had access to identifiable data or specimens for research purposes. Written informed consent was obtained from all adults and minors’ legal guardians at the interview.

## Results

### Study population

Prior to the COVID-19 pandemic, we aimed to collect data from 760 patients in 46 clusters. Due to COVID-19 TB service disruptions, 612 consenting patients were recruited in 34 of 46 clusters (12 clusters were considered non-eligible or not accessible due to the COVID-19 pandemic and TB service disruptions). Nine patients were ineligible and therefore excluded, hence a total of 603 participants were included in the analysis (i.e. 79.3% of the original sample size). Sixty-five (11%) enrolled TB patients had DR-TB and 538 (89%) had DS-TB disease. Three hundred seventy-one (61.5%) were male, and 60.8% belonged to the age group 25-54 years old **(Table 1).** These numbers are in line with the national TB epidemiology (among all nationally notified TB cases in 2020, 68% were male and 3% were children) [3, 6].

Education levels varied from 26.5% participants with 4-7 years of schooling to 8.5% with high education (studied 15 years or more). Four hundred and six respondents (67.4%) were formally employed prior to TB diagnosis, higher than the 57.9% national employment rate [39]. The average household size in the sample was 3.4 persons, slightly above the national average of 3.3 persons.

Although 100% of the population in Brazil is covered by the Unified Health System (SUS) [32], some people choose to pay for a private supplemental health plan. Among the study participants, 100 (16.6%) had voluntary private health insurance. Three hundred seventy-one (61.5%) respondents reported not having any type of social protection assistance (such as family allowance, sick pay, paid leave, emergency aid, or continued cash benefit).

Patients most frequently accessed TB care in Public Hospitals (28.0%) and Family Health units (18.9%). Only 73 (12.1%) received care in a private health service, and 41 (6.8%) in an Urgent and Emergency Care Unit. Three hundred twenty-five patients (53.9%) were interviewed in PHC and 278 (46.1%) in SHC facilities.

The interviews were carried out at two stages due to the COVID-19 pandemic. Collections initially took place from September 2019 to February 2020. During this period, 180 people (29.9%) were interviewed in 9 clusters. When interviews resumed, 423 people (70.1%) were interviewed from August to December 2020 and in April 2021 in 29 clusters (**Table 1)**. Forty-six cities were planned for data collection, four clusters did not have any patients at all and had to be dropped.

### Clinical characteristics of participants and use of health services

Most interviewed patients were registered as new TB cases (82.9%). Three hundred eighty-one patients (63.2%) were interviewed during the continuation phase of TB treatment. Among all interviewed patients, 82.9% had pulmonary TB (PTB), and 10.0% were co-infected with HIV. These proportions were 87% and 11% respectively at the national level in 2019 [2]. Four hundred thirty-two patients (71.6%) did not report other comorbidities, such as diabetes, hepatitis, and kidney disease.

Most respondents (85.7%) had not been hospitalized due to their TB. Four hundred and three patients (66.8%) used self-administered medication, and 81 (13.4%) were monitored by directly observed therapy (DOT) **(Table 2).**

### Patient’s care seeking pathway: Assessing patient’s time loss

During a TB episode, interviewed patients reported losing on average 79.9 (95%CI: 62.7–97.1) hours for diagnosis and treatment. This average reflects a mix of DR-TB patients losing on average 249.0 (95%CI: 115.6–382.4) hours and DS-TB patients losing on average 59.4 (95%CI:49.6–69.3) hours. Detailed breakdown of the time loss is presented in **Table 3.**

**Table 3:** Hours lost seeking or accessing TB care. Brazil national TB patient cost survey, 2019-2021.

Categories of time loss by patient or caregiver	TB patients (first-line treatment) (N = 538)	DR-TB patients (N = 65)	Total (N = 603)
**Pre-diagnosis by patient**			
Mean (95%CI)	16.0 (7.6, 24.3)	4.5 (-0.1, 9.2)	15.5 (7.5, 23.5)
Median (IQR)	3.4 (1.3, 6.5)	3.5 (1.4, 5.6)	3.4 (1.3, 6.5)
**DOT by patient**			
Mean (95%CI)	54.6 (43.6, 65.5)	181.3 (87.2, 275.4)	71.3 (54.6, 88.1)
Median (IQR)	39.0 (25.7, 67.6)	125.7 (77.4, 232.7)	39.0 (25.7, 77.9)
**Drug pick-up by patient**			
Mean (95%CI)	17.3 (13.8, 20.8)	108.9 (21.6, 196.2)	27.2 (17.2, 37.2)
Median (IQR)	8.6 (2.2, 19.5)	26.0 (0.8, 90.9)	8.6 (2.1, 25.7)
**Clinic visits by patient**			
Mean (95%CI)	13.0 (9.9, 16.2)	84.0 (30.2, 137.7)	20.7 (14.1, 27.2)
Median (IQR)	5.0 (1.8, 11.2)	19.3 (3.5, 49.1)	5.4 (1.9, 13.3)
**Total hours lost by patient**			
Mean (95%CI)	59.4 (49.6, 69.3)	249.0 (115.6, 382.4)	79.9 (62.7, 97.1)
Median (IQR)	25.5 (10.3, 60.3)	109.7 (39.1, 233.8)	28.8 (11.0, 73.4)
**Total hours lost by caregiver**			
Mean (95%CI)	6.4 (3.6, 9.1)	45.0 (7.1, 82.9)	10.5 (5.7, 15.3)
Median (IQR)	0.0 (0.0, 1.0)	0.0 (0.0, 0.0)	0.0 (0.0, 0.8)

**TB: Tuberculosis:** SD: standard deviation; IQR: Inter-quartile range; DR-TB: drug-resistant TB; 95% CI: 95% confidence interval

### Costs incurred by TB patients and their households

**Fig 2:**
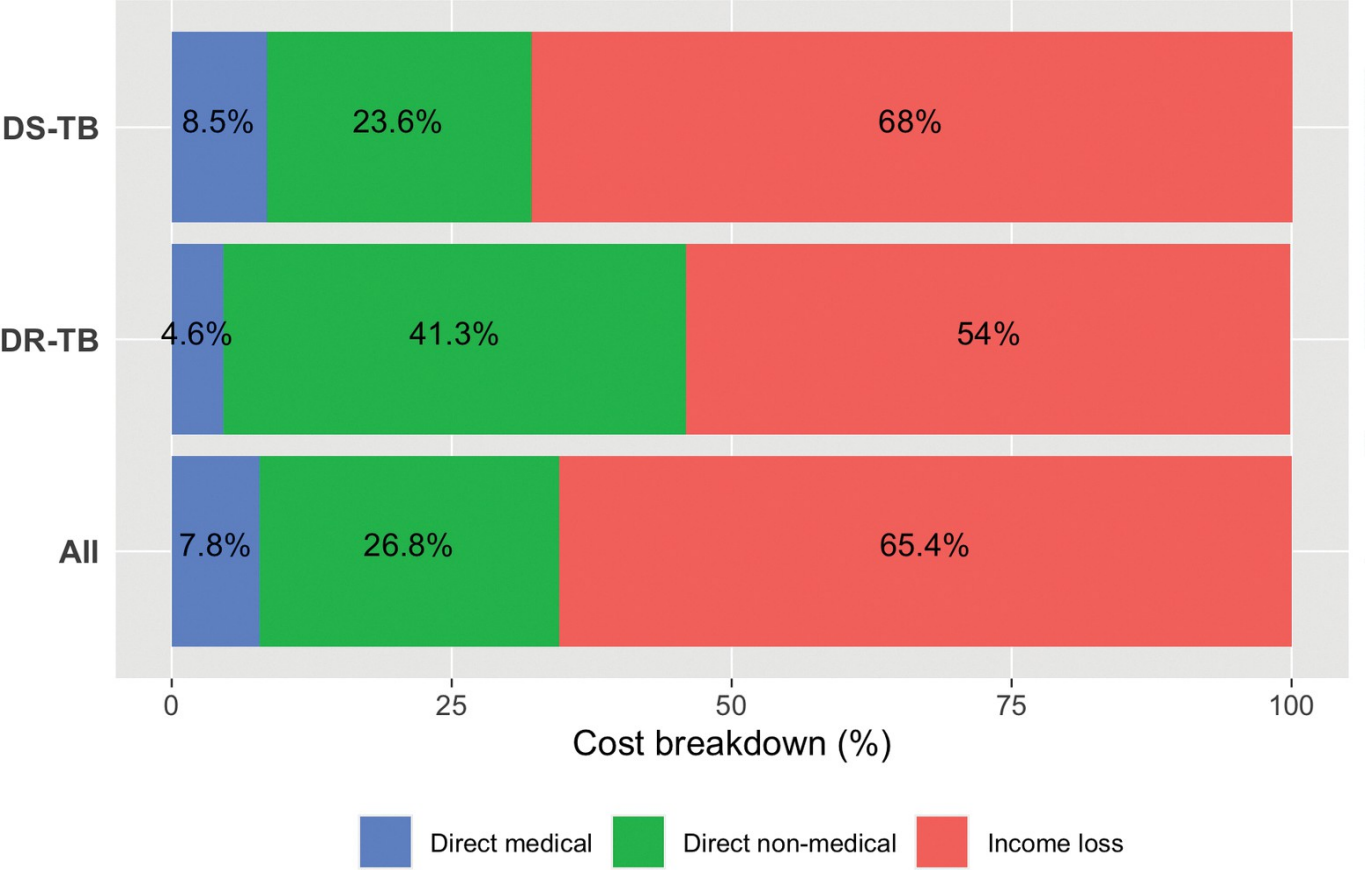
Distribution of total costs during TB episode, by cost category. Brazil National TB Patients Cost Survey, 2019–2021.

The estimated mean cost incurred by TB-affected households over an episode of TB was US$ 1,573.4 (95%CI: 1,361.8–1,785), including pre-diagnosis and post-diagnosis direct and indirect costs. Mean medical, non-medical, and indirect costs were US$122.1 (95%CI: 90–154.2), US$421.8 (95%CI: 332.6–510.9) and US$1,029.6 (95% CI: 834.0–1,225.1) respectively. Mean direct medical cost was higher among DR-TB patients (US$46.8, 95% CI: 35.4–59.1) compared to DS-TB patients (US$35.4, 95% CI: 31.2–39.7) before diagnosis and lower in the post-diagnosis period (US$76.2, 95%CI: 27.4–125.0 and US$86.5, 95% CI: 50.6–122.4, respectively). In the post-diagnosis period, the main driver of costs were direct non-medical costs including food supplements (US$317.6, 95% CI: 232.7–402.6) and travel for clinic and DOT visits (US$79.2, 95% CI: 61.9–96.5), followed by direct medical costs (US$85.5, 95% CI: 54.3–116.5). Indirect costs (loss of income) were higher among the households of DR-TB patients (US$1,437.2, 95% CI: 889–1985.3) compared to the households of DS-TB patients (US$980.3, 95% CI: 790.6 – 1,170.1) **(Fig 2, Table 4).**

**Table 4:** Estimated total costs borne by TB-affected households (HH). Brazil national TB patient cost survey, 2019-2021. Mean US$ [95% CI].

Cost category		HH of TB patients (first-line treatment) (N = 538)		HH of DR-TB patients (N = 65)		Total (N = 603)	
		Mean	CI	Mean	CI	Mean	CI
**Pre-diagnosis**	Medical	35.4	(31.2–39.7)	46.8	(34.4–59.1)	36.7	(32.8–40.6)
	Non-medical	5.4	(4.4–6.4)	5.3	(4.2–6.4)	5.4	(4.5–6.3)
**Post-diagnosis**							
	Medical	86.5	(50.6–122.4)	76.2	(27.4–125.0)	85.4	(54.3–116.5)
	Non-medical						
	Travel	65.7	(51.7–79.8)	190.7	(116.5–264.9)	79.2	(61.9–96.5)
	Accomodation	3.5	(2.0–4.9)	6.6	(-2.1–15.3)	3.8	(2.1–5.5)
	Food	11.9	(7.4–16.5)	47.5	(22.8–72.2)	15.8	(9.8–21.7)
	Nutrition supplement	253.4	(185.7–321)	849.5	(518.8–1180.2)	317.6	(232.7–402.6)
**Subtotal**							
	Medical	122.0	(85.6–158.3)	123.0	(76.0–169.9)	122.1	(90.0–154.2)
	Non-medical	339.9	(271.9–407.9)	1099.6	(773.2–1426.1)	421.8	(332.6–510.9)
	Indirect costs (income loss)	980.3	(790.6–1170.1)	1437.2	(889.0–1985.3)	1029.6	(834.0–1225.1)
**Total**							
**Total cost (output approach)**		1442.2	(1235.9 -1648.4)	2659.7	(2045.3–3274.2)	1573.4	(1361.8 – 1785.0)

95% CI: 95% confidence interval

### Income loss and poverty level changes

At the onset of the TB disease, average monthly individual income was higher among DS-TB patients (US$ 401.6 (95% CI: 364.8–438.3)) compared to patients with DR-TB (US$ 334.4, (95% CI: 274.8–393.9)). The average individual income was US$ 409.7 (95% CI: 369.2–450.3) **(Table 5).**

**Table 5:** Monthly self-reported income and poverty level among study participants. Brazil national TB patient cost survey, 2019-2021.

Income category	TB patients (first-line treatment) (N = 538)	Patients with drug-resistant TB (N = 65)	Total (N = 603)
**Individual income at onset of TB**	409.7 (369.2, 450.3)	334.4 (274.8, 393.9)	401.6 (364.8, 438.3)
**Household income at onset of TB**	617.1 (561.7, 672.5)	449.1 (374.6, 523.7)	599.0 (548.8, 649.2)
**Individual income at interview**	422.9 (378.0, 467.9)	327.6 (266.1, 389.0)	412.7 (372.0, 453.3)
**Household income at interview**	502.6 (444.7, 560.5)	331.0 (277.9, 384.0)	484.1 (432.0, 536.2)
**Patient was main income earner at onset of TB**			
equal*	24 (4.5%)	8 (12.3%)	32 (5.3%)
no	255 (47.5%)	25 (38.5%)	280 (46.5%)
yes	258 (48.0%)	32 (49.2%)	290 (48.2%)
Households below poverty line** **at onset of TB**	4.3% (2.5%-6.5%)	4.6% (0.83%-11.2%)	4.3% (2.7%-6.3%)
Households below poverty line** **at interview**	8.7% (6.1%-11.7%)	29.2% (19.5%-40.0%)	10.9% (8.1%-14.2%)

*All adults in the household earned equally

** Defined as US$ 1.90 PPP

The average monthly household income at the onset of the disease was US$ 599.0 (95% CI: 548.8–649.2), with DS-TB patients reporting higher household income (US$ 617.1 (95% CI: 561.7–672.5)) than DR-TB patients (US$449.1 (95% CI: 374.6–523.7). The average monthly household income after diagnosis dropped to US$484.1 (95%CI: 432.0–536.2), which means an average loss of US$115 dollars per household **(Table 5)**.

The proportion of tuberculosis-affected households with self-reported income below the poverty line doubled from 4.3% (95% CI: 2.7 – 6.3) prior to TB to 10.9% (95% CI: 8.1 – 14.2) at the time of the interview. For DR-TB affected households, the proportion rose from 4.6% (95% CI:0.83 – 11.2) prior to TB to 29.2% (95% CI: 19.5 – 40.0) at the time of the interview. We did not observe any difference in income loss and poverty level changes between patents interviewed before and during COVID-19 pandemic.

### Coping mechanisms, social consequences, and changes in employment status during TB episode

The social impact of TB and mechanisms adopted by households in Brazil to deal with the economic burden imposed by the disease are shown in **Table 6**. Thirty-two percent of enrolled TB patients had to resort to either borrowing or selling assets to cope with TB, and 54.9% reported experiencing some sort of social impact (i.e., social exclusion, food insecurity, job losses, interrupted schooling, or divorce). Thirty-five percent reported social exclusion. Thirty-seven percent and 7.7% of patients reported becoming poorer or much poorer, respectively, since the onset of the first TB symptoms.

**Table 6:** Coping mechanisms, social consequences and financial impact due to TB care by drug resistance status. Brazil national TB patients cost survey, 2019–2021.

Category	TB patients (first-line treatment) (N = 538)	DR-TB patients (N = 65)	Total (N = 603)
**Coping mechanism**			
Loan	151 (28.1%)	21 (32.3%)	172 (28.6%)
Asset sale	38 (7.1%)	11 (16.9%)	49 (8.1%)
Any of above	169 (31.5%)	24 (36.9%)	193 (32.1%)
**Social consequences**			
Social exclusion	183 (34.1%)	30 (46.2%)	213 (35.4%)
Food insecurity	30 (5.6%)	8 (12.3%)	38 (6.3%)
Job loss	116 (21.6%)	17 (26.2%)	133 (22.1%)
Interrupted schooling	10 (1.9%)	2 (3.1%)	12 (2.0%)
Divorce	6 (1.1%)	3 (4.6%)	9 (1.5%)
Any of above	290 (53.9%)	41 (63.1%)	331 (54.9%)
**Perceived financial impact**			
Much poorer	39 (7.3%)	7 (10.8%)	46 (7.7%)
Poorer	192 (35.9%)	28 (43.1%)	220 (36.7%)
Unchanged	302 (56.4%)	30 (46.2%)	332 (55.3%)
Richer	1 (0.2%)	0 (0.0%)	1 (0.2%)
Much richer	1 (0.2%)	0 (0.0%)	1 (0.2%)

Of the 603 survey participants, 232 (38.5%) reported receiving some benefits from the Brazilian government; among them 31 DR-TB and 201 DS-TB patients. In total, there were 238 benefit occurrences (as some patients received more than one benefit) including 38 (16%) from Bolsa Familia Programme, 52 (22%) sick pays, 124 (52%) COVID-19 emergency social assistance benefits, and 24 (10%) other benefits such as Continuous Cash Benefit and utility vouchers. Most TB patients received the COVID-19 emergency social assistance in the amount of 116 dollars per month. An analysis by income quintile showed that wealthier patients received less social protection (22.7%) compared to poorest patients (43.0%).

**Fig 3:**
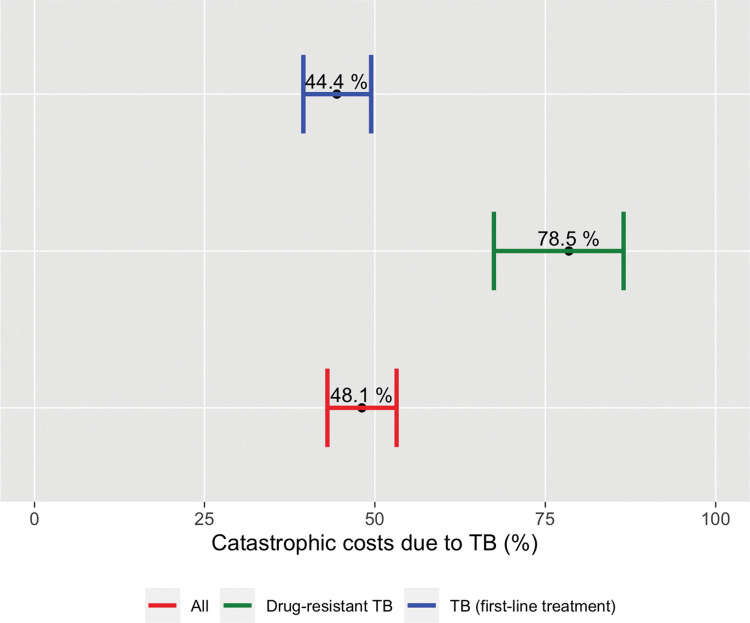
Changes in employment status of enrolled TB patients before and during TB episode. Brazil national TB patients cost survey, 2019–2021.

The impact of TB on employment status is highlighted in **Fig 3**. Employment of any form among patients dropped from 68% to 48% with both formal and informal employment levels dropping by 10%, presumably due to lower licensure or sick leave protection. Twenty-two percent (22%) of patients reported losing their jobs and became unemployed during TB treatment **(Table 6)**. No differences in patient’s employment status change due to TB was observed between patients interviewed before and during COVID-19 pandemic.

## Catastrophic costs due to TB and risk factors for incurring catastrophic costs

**Fig 4:**
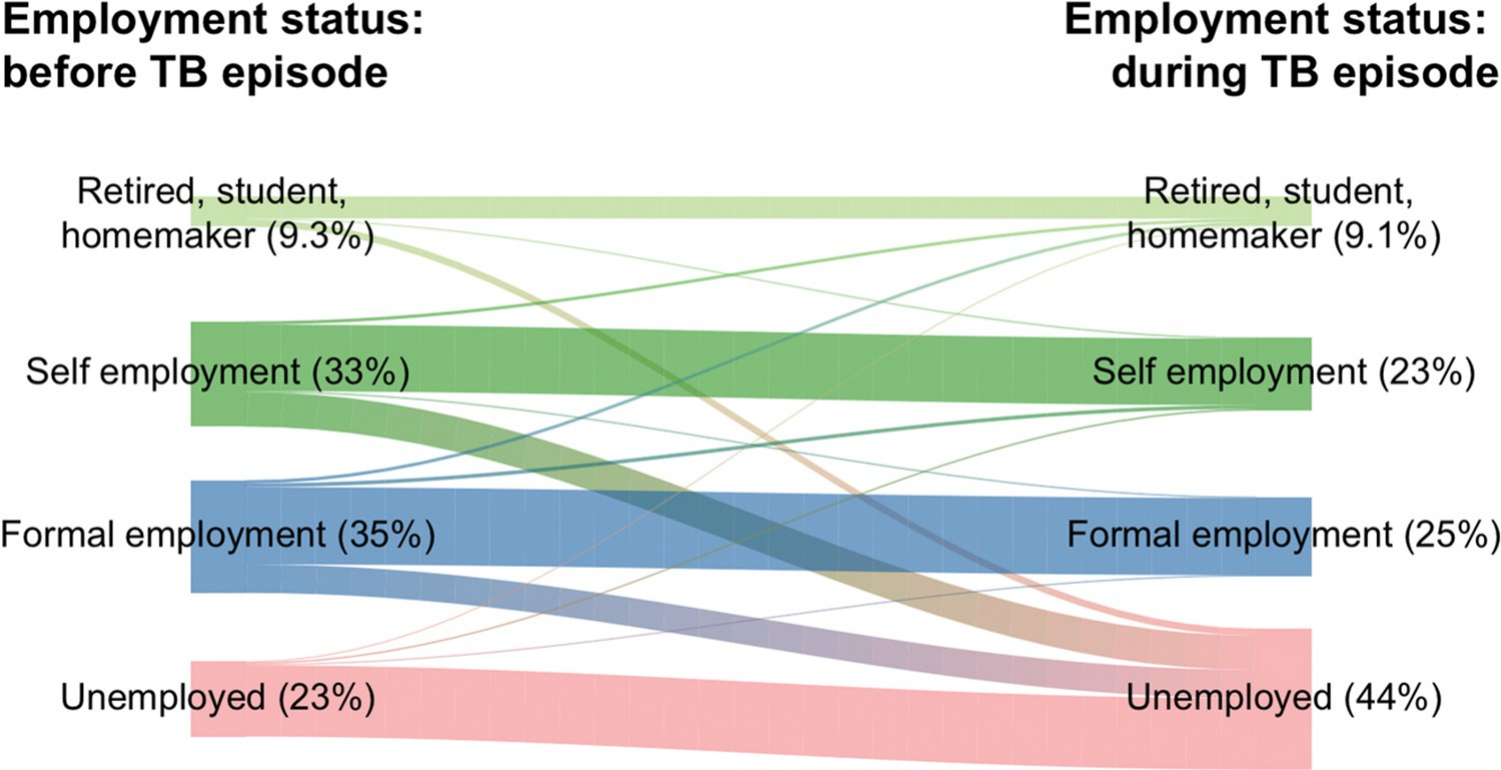
Estimated proportions of TB-affected households experienced catastrophic costs* (using main approach and a threshold of 20%). Brazil national TB patients cost survey, 2019–2021.

The proportion of TB-affected households facing costs >20% of household income due to TB in Brazil in 2019-2021 was 48.1% (95% CI: 43.0–53.2). This proportion ranged from 44.1% (95% CI: 39.5–49.5) for DS-TB to 78.5% (95% CI: 67.5–86.5) for DR-TB patients/households **(Fig 4).**

After adjusting for potential confounders and covariates in multivariate analysis, HIV-positive status (aOR = 3.0, 95% CI:1.1–8.6, p = 0.037) and self-employment (aOR = 2.7, 95%CI:1.1–6.5, p = 0.030) increased the chance of incurring catastrophic costs; high education level was a protection factor (aOR = 0.1, 95%CI:0.0–0.9, p = 0.035) **(Table 7)**

**Table 7:** Univariate and multivariate analysis of catastrophic costs predictors. Brazil national TB patients cost survey, 2019–2021.

**Characteristic**	**Category**	**OR-univariate**	**95% CI**	**p-value**	**OR-multivariate**	**95% CI**	**p-value**
Age group	<15	Ref	NA	NA			
	15-24	0.4	(0.1-1.6)	0.208			
	25-34	0.8	(0.2-3.4)	0.778			
	35-44	0.7	(0.2-2.8)	0.6			
	45-54	0.8	(0.2-3.5)	0.788			
	55-64	0.5	(0.1-2.2)	0.346			
	>65	0.3	(0.1-1.2)	0.089			
Sex	Female	Ref	NA	NA	Ref	NA	NA
	Male	1.3	(0.9-1.7)	0.107	0.8	(0.4-1.7)	0.533
Drug resistance status	Drug sensitive	Ref	NA	NA	Ref	NA	NA
	Drug-resisitant TB	4.6	(2.5-8.2)	<0.001	3.7	(0.4-36)	0.262
Type of TB	Pulmonary TB	Ref	NA	NA			
	Extra pulmonary TB	0.9	(0.7-1.3)	0.659			
Treatment delay > 4 weeks	No	Ref	NA	NA	Ref	NA	NA
	Yes	0.6	(0.4-1.1)	0.093	0.8	(0.4-1.4)	0.449
Retreatment status	No	Ref	NA	NA	Ref	NA	NA
	Yes	1.4	(0.9-2.4)	0.167	0.5	(0.1-1.9)	0.305
Hospitalization during TB episode	No	Ref	NA	NA			
	Yes	1.2	(0.7-1.8)	0.542			
TB treatment mode	No DOT	Ref	NA	NA			
	DOT	1	(0.7-1.5)	0.89			
**Characteristic**	**Category**	**OR-univariate**			**Characteristic**	**Category**	**OR-univariate**
HIV Status	Negative	Ref	NA	NA	Ref	NA	NA
	Positive	1.1	(0.6-1.9)	0.753	3	(1.1-8.6)	0.037
Education level	Less then Primary education	Ref	NA	NA	Ref	NA	NA
	Primary education	0.6	(0.3-1.1)	0.12	0.6	(0.2-2.0)	0.408
	Secondary or higher	0.2	(0.1-0.5)	0.001	0.1	(0.0-0.9)	0.035
Employment type	Unemployed	Ref	NA	NA	Ref	NA	NA
	Employed (formal)	1.1	(0.7-1.7)	0.836	0.6	(0.3-1.5)	0.3
	Self employed	2.3	(1.5-3.7)	0.001	2.7	(1.1-6.5)	0.03
	Student/Retired/Others	1	(0.5-2.1)	0.967	1.9	(0.6-5.5)	0.247
Patient was main income earner	No	Ref	NA	NA			
	Yes	1.2	(0.8-1.7)	0.306			
	Equal	1.2	(0.6-2.6)	0.655			
Insurance	No	Ref	NA	NA	Ref	NA	NA
	Any Insurance	0.5	(0.3-0.8)	0.007	0.5	(0.1-1.7)	0.236
Social protection support	No	Ref	NA	NA	Ref	NA	NA
	Yes	1.8	(1.3-2.6)	0.001	0.8	(0.4-1.6)	0.472
Income quintile	5th	Ref	NA	NA	Ref	NA	NA
	4th	1.9	(1.0-3.9)	0.064	1.4	(0.4-4.3)	0.585
	3rd	1.4	(0.7-2.7)	0.291	1.5	(0.6-3.8)	0.379
	2nd	2.5	(1.5-4.4)	0.001	2.7	(1.0-7.2)	0.046
	1st (lowest)	1.4	(0.8-2.7)	0.267	0.8	(0.2-3.0)	0.694
Household size	≤5	Ref	NA	NA			
	5+	1.1	(0.8-1.7)	0.557			
Data collection period	Before COVID-19	Ref	NA	NA			
	During COVID-19	1	(0.7-1.4)	0.812			

TB: tuberculosis; 95% CI: 95% confidence interval

**Fig 5:**
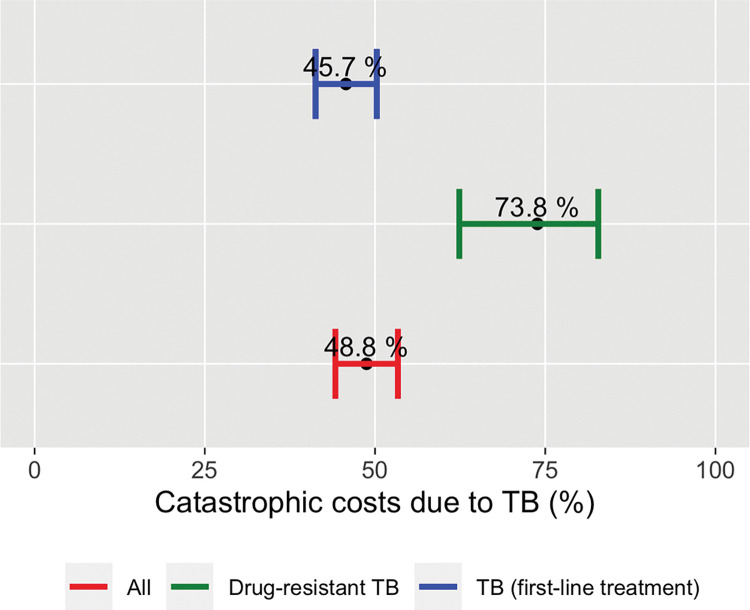
Estimated proportions of TB-affected households (HH) experienced catastrophic costs (using alternative measure of ability to pay). Brazil national TB patients cost survey, 2019–2021. Costs are estimated using the output approach: HH Income (PRE-TB) - HH Income (POST-TB) + DIRECT COSTS / HH assets-based approach with a threshold of 20%. This approach is for sensitivity analysis purposes to explore the impact of altering the measure of ability to pay in the resulting End TB indicator proportion.

**Fig 6:**
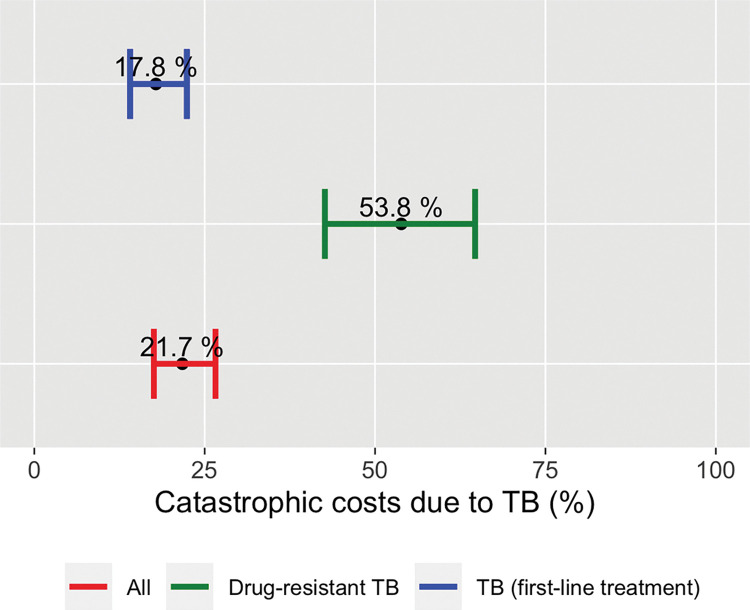
Estimated proportions of TB-affected households experienced catastrophic costs (using an alternative measure for indirect cost calculation, the human capital approach). Brazil national TB patients cost survey, 2019–2021. Costs are estimated using the output approach: DIRECT COSTS + VALUATION OF TIME LOSS (human capital approach) / HH Income (PRE-TB) with a threshold of 20%. This approach is for sensitivity analysis purposes to explore the impact of altering the measure of indirect cost measurement in the resulting End TB indicator proportion.

In the sensitivity analyses we explored two alternative methods to measure the proportion of households experiencing catastrophic costs and also explored the impact of altering the threshold that defines costs as catastrophic. In addition to the globally monitored 20%, we also explored 10%, 25% and 40% (**Table 8**). As a first alternative, we estimated indirect costs measuring household income change (using the same output approach), but income was measured based on asset ownership. As a second alternative method, we estimated indirect costs using a valuation of time loss (human capital approach) at the self-reported individual hourly wage and compared it against self-reported household income prior to the disease. At a threshold of 20%, 48.8% (95% CI: 44.2–53.4) and 21.7% (95% CI: 17.5–26.6) households incurred catastrophic costs in the two alternatives respectively **(Figs 5 and 6).** Finally, we also evaluated the proportion of TB-affected households incurring in direct medical costs that amounted to 10% or more of their self-reported household income, which resulted in 3.98% (95% CI: 2.59–6.08) amongst all patients (**Table 8**).

**Table 8:** Proportion of households experiencing costs above various thresholds of household income.

Sensitivity threshold	%	CI
Total costs borne by TB-affected households per TB episode		
20% (End TB Indicator)	48	(43-53)
25%	40	(35-45)
30%	32	(27-37)
35%	26	(43-53)
40%	19	(15-24)
**Direct medical costs** **borne by TB-affected households per TB episode**		
10%	3.8	(2.4-5.9)
20%	1.8	(1.0-3.2)
25%	1.8	(1.0-3.2)
30%	1.2	(0.23-2.5)
35%	1	(0.41-2.4)

## Discussion

This study is the first effort to establish a baseline measure of the End TB catastrophic cost indicator in Brazil. The study aimed to determine the baseline proportion of Brazilian households incurring costs exceeding 20% of their annual income due to TB. We found that 48.1% of TB-affected households participating in the study experienced high costs above 20% of their annual income when seeking care and treatment services due to TB.

These high costs affect TB patients contributing to worsening their already vulnerable situation and imposing unnecessary economic burden to their households. The results of our study are in line with the findings of the two previous sub-national studies in Brazil. The first one estimated that 46% of evaluated DS-TB households experienced catastrophic costs due to TB [23]. In our study we found that 44.4% DS-TB-affected households and 78.5% DR-TB-affected households experienced catastrophic costs due to the disease. Our result for DR-TB patients and their households is consistent with the findings of the Rio de Janeiro’s study where 68% of DR-TB patients experienced catastrophic costs due to TB [24]. Based on these results social protection measures could be specifically evaluated for DR-TB patients.

The higher number of enrolled males is in line with the previous studies and national epidemiology. Historical data reported by the Brazilian National Tuberculosis Control Program to WHO shows that 46,130 (69.0%) of TB cases occurred in males from 2011 to 2020 [4, 5].

Most of the enrolled patients had pulmonary TB, were being treated first time with the first line TB drugs and were classified as new TB cases. In this study, 62 TB patients (10.3%) did not know their HIV status (compared to 15% nationally in 2020) [6], and 60 patients (10.0%) were co-infected with HIV. In 2020, 80% of all notified new and relapse TB cases knew their HIV status in Brazil, and 8.4% of new TB cases were HIV positive [5]. Another multicentre study in Brazil found that 13% of the population were not tested for HIV [40]. Routine HIV testing is one of the key guidelines of the clinical TB protocol in Brazil as the TB-HIV co-infection increases the probability of death for people affected by these diseases. Progress in implementing HIV testing among TB patients has improved over time from 51% in 2003 to 80% in 2020 when a slight drop in testing rate occurred compared to pre-COVID-19 83%. It would be essential for 100% of testing to be aimed for to reach End TB targets including with the positive indirect impact on one of the risk factors influencing the incidence of catastrophic costs in Brazil.

The mean cost incurred by TB-affected households over an episode of care was US$ 1,573.4, which is approximately 7 times higher than the minimum monthly wage in Brazil (US$ 213.2) [41]. Non-medical costs during TB treatment were substantial and driven by travel related to TB care (US$79.2), the costs of nutritional supplements outside of regular diet because of TB disease, e.g., vitamins as recommended by health care staff (US$ 317.6) and food (US$ 15.8) purchased during treatment, highlighting that even in a country with a universal health system, the expenditure due to TB can be high.

Our study results demonstrated that one-third of the study participants had to use different coping mechanisms to address the economic burden imposed by TB disease. Taking a loan or selling assets were options to compensate for the loss of income and high out-of-pocket expenses incurred by patients while accessing TB care. We also observed that more than half of the enrolled patients reported that TB somehow affected their social or private lives, with social exclusion and food insecurity (difficulty in purchasing food) being the most prevalent responses. Employment amongst TB-affected patients changed drastically with 22% patients reporting job losses during the episode (**Fig 4)**. These results are also in line with the findings of the previous sub-national studies in Brazil which highlighted job losses that often occurs amongst TB-affected patients and leads to a decrease in family income [41, 42].

More than one-third of the study participants received some type of social protection benefits from the Brazilian government (**Table 1**). The maximum amount received per patient over an episode of TB was US$ 213.2 per month, which represents a small increase in family income, considering that a basic monthly food basket in Brazil in the same period was equivalent to US$ 121,7. Cash transfers were provided during COVID-19 pandemic as an emergency social assistance measure. To be eligible for this support (US$ 116 per month), the total monthly family income had to be less than one-third of the minimum monthly wage (US$ 213.2). In our study, among 603 enrolled individuals, 124 (21%) had received COVID-19 cash transfers. Previous studies demonstrated that social protection benefits from the Brazilian government are important for vulnerable populations and positively impact the outcome of TB [43–45].

Most study participants reported becoming poorer or much poorer while on TB treatment. When measuring self-reported household income changes, we observed a decline from US$ 599.0 to US$ 484.1, an average loss of US$ 115 dollars across households **(Table 5)**. Most TB patients interviewed in this study reside in communities with severe problems of social determinants of health, such as inadequate housing, peripheral neighbourhoods with social vulnerability, high demographic density, inadequate working conditions, and difficulties accessing services; these findings were also consistent with the results from other studies [40].

During the second phase of data collection, the Brazilian government had begun providing social assistance to the population as part of COVID-19 impact mitigation interventions. The conditional cash grants offered by the Brazilian government were not included in our calculation of catastrophic costs, but we recognise that this may have affected the TB affected households’ income. We defined a control variable indicating whether the interview took place before or during the pandemic, and checked for significant differences in key variables. We did not observe any differences in socio-economic status, poverty level, cost estimates and income loss between patients interviewed before and during COVID-19 pandemic. These could be because receiving the emergency aid (including cash transfer) may not have induced immediate changes in TB affected households’ health seeking habits. Longitudinal analyses may be needed to further ascertain the changes that have happened.

## Research findings leading to change in policy and practice in Brazil

According to the data from the IBGE, unemployment in Brazil impacted 6.7 million people in 2014 and 12.6 million in 2019 (i.e., 90% increase over the 5-year period), with the unemployment rate increasing from 6.8% to 11.9%, respectively. At the same time, the country’s labour market also went through an accelerated process of precariousness, with an increase in the number of self-employed workers without labour guarantees.

Results from this study show that income and job losses are significant problems for the TB-affected households, and the challenges during the post-COVID-19 pandemic period could be even worse. In fact, recent modelling for Brazil (and other 15 countries) by WHO and partners suggests the impact of COVID on TB incidence and mortality will be much larger in 2021 and beyond, especially on TB mortality (+20%) in 2021 and TB incidence in 2022 [6]. Hence having cash transfer as a form of social assistance is vital for TB patients to ensure they can successfully complete their TB treatment and Brazil can progress towards eliminating TB as a public health issue [43]. During the pandemic, emergency aid was created by the Brazilian government for the most vulnerable through Decree No. 10,316, of April 7, 2020 demonstrating beneficial effects of cash transfers to vulnerable households. People who already received government aid were also covered with this added value, which provided an increase in the income of that household during the period of aid in the pandemic.

In Brazil, the SUS and SUAS programs are playing an important role in establishing policies that provide social protection and health assistance aimed at mitigating catastrophic costs for TB patients, however, this assistance could be insufficient because many conditionalities greatly limit the number of people who can access these programs [39, 46]. In July 2021, an agreement was signed between the SUS and the SUAS, primarily regarding inclusion of socially vulnerable people with HIV, viral hepatitis, leprosy, tuberculosis, and prevention of congenital syphilis in these programs. Although the agreement is only valid for the subsequent 6 months (i.e., until 2022), it is the first step towards protecting vulnerable households from catastrophic costs and achieving the End TB Strategy goals established by the WHO.

TB is perceived as a medical condition which is mostly curable, however our results demonstrate that TB has social, income, employment and poverty consequences as well as potential long-lasting effects and social sequelae that require a multisectoral response. The results of this study have been presented to the Ministry of citizenship (where the social protection programs are located) and health, including all the country’s tuberculosis program coordinators, and were also presented to the congress of the Brazilian society of tropical medicine, where policy translation was discussed. Lessons learned from this survey will be used to design measures to alleviate the burden of disease on TB patients and their families, and also inform subsequent national surveys to monitor the WHO End TB Strategy indicator of TB catastrophic costs in Brazil.

### Strengths and limitations

Our study had several limitations. First, the sample was calculated based on the 2017 notifications, available at the time of the protocol design. However, the project was implemented in 2019-2021, so some targeted clusters had fewer patients per stratum than was anticipated by the 2017 sampling frame. The second limitation was that the estimates for DR-TB patients (N = 65) were not adjusted for finite population, giving more conservative confidence bounds. Brazil had 701 and 653 patients on DR-TB treatment in 2019 and 2020 respectively [6]. Third, the COVID-19 pandemic, which started five months after initiation of data collection, affected all research operations and suspended data collection for several months. The severe second wave of pandemic in Brazil impacted all health services delivery and forced the data collection to be concluded with 79% of the expected sample size recruited. The fourth limitation is related to the data collection process during the COVID-19 pandemic which saw most data collected in specialised services and fewer than expected in PHC services. During this period, PHC services were reorganized following recommendations of municipal programs and included protocol safety measures for patients in the face of prioritization of care to COVID-19.

Another point that deserves to be highlighted is that TB-HIV-affected households and those without a formal job or self-employed had a greater chance of incurring catastrophic costs in 2019-2021. TB and HIV co-infection poses significant financial challenges to the individuals, whether due to drug interactions or because these patients are generally monitored in specialized centres far from their homes and workplaces [47]. Not having a formal employment contract proved to be a predictor for incurring catastrophic costs by TB-affected households, as TB patients lose workdays that compromise their income. Patients who are formally employed are usually entitled to paid sick leave but self-employed or informally employed are not.

Despite the limitations, this study valuably contributes to Brazil’s and WHO’s global monitoring of the End TB Strategy indicator. The proportion of TB-affected households facing costs >20% of household income or expenditure due to TB in Brazil is in line with the global (pooled) average from the surveys implemented since 2015 following WHO methodology. Amongst low-and-middle-income countries reporting this proportion to WHO in 2021, the proportion of the households facing catastrophic costs in Brazil is higher than in 9 other countries (El Salvador, Lesotho, Kenya, Papua New Guinea, Benin, Fiji, The Philippines, Indonesia and Tanzania) but lower than in Uganda, Burkina Faso, DR Congo, Myanmar, Lao, Viet Nam, Ghana, Mongolia, Nigeria, Timor-Leste and Solomon Islands. This difference can be explained by multiple factors including varying geographic, health system, universal health care, and economic profiles in these countries [10, 12, 14, 15, 17].

## Conclusion

This study showed that almost one in two TB-affected households in Brazil incurred catastrophic costs due to TB in 2019, with significant income losses, a similar situation to latest global estimates (47% (95% CI:33-61%). With the poverty levels doubling after diagnosis, access to care could be compromised and treatment outcomes impacted.

A better alignment between the SUS and SUAS programs can alleviate the financial and economic burden experienced by households affected by TB disease in Brazil. The expansion of existing social protections and creation of new interventions to support TB patients could help mitigate the financial burden and reduce the proportion of households that experience catastrophic costs associated with TB disease in the country.

## Supporting information

S1 File Portuguese translation of questionnaire. (DOCX)

S2 File (PDF)
