# Supplementary material for: The economic burden of households affected by tuberculosis in Brazil: First national survey results, 2019-2021
Source: PLoS One. 2023 Dec 13;18(12):e0287961. doi: 10.1371/journal.pone.0287961 (PMC10718450; doi:10.1371/journal.pone.0287961)
Supplement: S1 File — (DOCX) [file pone.0287961.s001.docx]

**Anexo G**

Questionário Paciente TB Custos

Questionário de pesquisa para o Brasil

**Parte I. Informações do paciente obtidos do cartão de tratamento TB antes da entrevista**

| **ID_______________________** | |  | | | | | |
| --- | --- | --- | --- | --- | --- | --- | --- |
| Questão | | Categorias de respostas (círculo apropriado número ou preenchimento resposta na linha de resposta) | | | | **Ação para entrevistador**  as perguntas na parte 1 não são parte da entrevista e devem ser preenchidas antes da entrevista | |
| 1. Data da entrevista | | (Dia/mês/ano)……/……/……… | | | |  | |
| 1. Selecione um município | | 1. ANANINDEUA 2. APARECIDA DE GOIANIA 3. ARAGUAINA 4. ARAPIRACA 5. ATIBAIA 6. BALNEARIO CAMBORIU 7. BELO HORIZONTE 8. CAMARAGIBE 9. CANELINHA 10. DUQUE DE CAXIAS 11. FORTALEZA 12. GOIANIA 13. ITACOATIARA 14. ITAREMA 15. JOAO PESSOA 16. JOINVILLE 17. JUIZ DE FORA 18. MANAUS 19. NOVA IGUACU 20. OLINDA 21. PACAJA 22. PORTO ALEGRE 23. POTIM 24. RECIFE 25. RIO DAS OSTRAS 26. RIO DE JANEIRO 27. SALVADOR 28. SAO GONCALO 29. SAO PAULO 30. SOBRAL 31. TERESOPOLIS | | | |  |  |
| 1. Selecione Região | | 1. CENTRO OESTE  2. NORDESTE  3. NORTE  4. SUDESTE  5. SUL | | | |  |  |
| 1. Selecione o local da entrevista (nome de instalação) | | ……………………………. | | | |  |  |
| 1. Nome do entrevistador | | ……………………………. | | | |  |  |
| 1. Tipo de instalação | | 1. Atenção primária  2. Especializada | | | |  | |
| 1. Categoria de instalações de tratamento | | 1. Unidade de saúde da família  2. Unidade básica de saúde  3. Unidade de referência para tuberculose  4. Unidade de cuidados de emergência  5. Hospital  6. ONGs  7. Serviço privado  8. Outros | | | | A instalação de "tratamento" é o local onde está guardado o cartão de tratamento do paciente. | |
| 1. Paciente ID |  | | | |  | | |
| 1. Sexo | 1. Masculino  2. Feminino | | | |  | | |
| 1. Idade do paciente: se < 1 ano de idade | ______ anos  ______ meses | | | | Se o paciente estiver abaixo de um ano de idade, nós dividimos o número de meses de idade do paciente, sobre o número de meses em um ano (12) | | |
| 1. Data do diagnóstico | (Dia/mês/ano) ……/……/……… | | | |  | | |
| 1. Local do diagnóstico | 1. Unidade de saúde da família  2. Unidade básica de saúde  3. Unidade de referência para tuberculose  4. Unidade de cuidados de emergência  5. Hospital  6. ONGs  7. Serviço privado  8. Outros | | | |  | | |
| 1. Tipo de TB | 1. Pulmonar. Bacteriologicamente confirmada.  2. Pulmonar. Não-confirmado bacteriologicamente.  3, Extrapulmonar | | | |  | | |
| 1. No tratamento de MDR-TB | 1. Sim  2. Não | | | |  | | |
| 1. Duração total do tratamento planeado desde início (meses): fase intensiva | (_____ meses) | | | | Se o paciente está na fase de continuação, a duração para a fase intensiva deve ser relatada como reais meses nessa fase. | | |
| 1. Duração total do tratamento planeado desde início (meses): fase de continuação | (_____ meses) | | | | Se o paciente está numa fase intensiva. a duração para a fase de continuação deve ser relatada como reais meses nessa fase. | | |
| 1. Grupo de registo de tratamento | Não MDR  1. 1ª linha, nova  2. 1ª linha, recidiva  3. 1ª linha, retratamento após perda de seguimento  4. Resistência de uma ou mais drogas, especificar:……………..  MDR  5. MDR, nova (MDR inicial)  6. MDR, recidiva  7. MDR, retratamento após perda de seguimento  8. MDR, retratamento após a falha do primeiro tratamento com drogas de 1ª linha  9. MDR, retratamento após a falha do regime de retratamento com drogas de 1ª linha  10. Outros, especificar: ……………………… | | | | Se "Outro" (resposta 17), excluir do estudo | | |
| 1. Data de início do atual tratamento de TB | (Dia/mês/ano) ……/……/……… | | | |  | | |
| 1. O paciente está atualmente em fase de tratamento intensivo ou continuação? | 1. Concluiu a fase intensiva, ___dias da fase  2. Fase de continuação, ___dias da fase concluída | | | | Se o paciente tiver concluído a menos de 14 dias da fase atual de tratamento, excluir, ou adiar a entrevista. Entrevista ocorre depois que completaram um mínimo de 14 dias. Fase intensiva para regimes de MDR-TB é o período de tratamento inicial, que inclui uma droga injetável (geralmente 4 a 8 meses). | | |
| 1. Quantos dias desta fase foi concluída? |  | | | |  | | |
| 1. Status de HIV (conforme indicado no cartão de tratamento) | 1. Positivo.  2. Negativo  3. Não testado  4. Desconhecido | | | |  | | |
| 1. Paciente hospitalizado no momento da entrevista? | 1. Sim  2. Não | | | |  | | |
| 1. Data, quando o paciente foi admitido | (Dia/mês/ano) ……/……/……… | | | |  | | |
| 1. Moeda usada na entrevista: | ……….. | | | | Tipo de relatório da moeda. por exemplo, USD | | |
| **Lista de verificação de quais partes do questionário são para preencher para categorias diferentes de tratamento** | | | | | | | |
| **Categoria de tratamento e fase de tratamento, no momento da entrevista** | | | **Questionário parte IV (ticar quando cheio)** | **Parte do questionário V (ticar quando cheio)** | | | **Supervisor check** |

| **Parte II - custos antes do atual tratamento de TB (preenchido para os casos em fase intensiva apenas)** | | |
| --- | --- | --- |
| **Despesas do próprio bolso, reembolsos e perda de tempo, antes e durante o diagnóstico de TB (antes do início do tratamento de TB)** | | |
| **Questão** | Categorias respostas (marque todas que se aplicam ou preencher a resposta na linha de resposta) | **Instruções e as ações para o entrevistador** |
| 1. Para este episódio de TB, quantas semanas antes de começar o tratamento para tuberculose você experimentou sintomas para este episódio de TB? | Semanas antes do início do tratamento:________ | Muitas vezes há um problema que define os “sintomas da tuberculose”, pois os pacientes não sabem que a tosse, a febre ou a perda de peso que inicia o processo de procura de cuidados tem alguma relação com a tuberculose. Portanto, certifique-se de começar com perguntas abertas sobre os sintomas que elas experimentaram nos estágios iniciais da doença e depois perguntar quando esses sintomas ocorreram pela primeira vez, quando pioraram e começaram a preocupar o paciente e levaram o paciente a procurar atendimento. Construa uma linha do tempo de eventos, seja a partir dos primeiros sintomas, ou comece com o tempo de diagnóstico de TB e trabalhe de trás para frente. Use o calendário adaptado localmente com os principais eventos sazonais com os quais o paciente pode se relacionar e usar como ponto de referência para o tempo. Use esta linha do tempo para mapear as datas de todos os episódios de busca de cuidados ou conselhos para ajudar a registrá-las nas respostas para a próxima pergunta (ver Pergunta 26). Você só deve investigar os sintomas clássicos de tuberculose da tosse, perda de peso, dor torácica e sudorese noturna e seu tempo se eles não foram relatados durante o questionamento aberto. |
| 1. Quanto dinheiro e tempo gastou para cada uma destas visitas antes de que foi diagnosticado com tuberculose, incluindo a visita quando você realmente recebeu seu diagnóstico? | Veja a tabela abaixo. e pergunte por cada item.  • Preencha uma linha por visita;  • Para todos os que não se aplicam, marque / selecione NA  • Adicione mais linhas se mais visitas forem feitas antes do diagnóstico de TB!  Explicação dos títulos da tabela:  Visitas: inclui visitas ambulatoriais e hospitalizações. Deve ser preenchido em ordem cronológica, primeira visita = visita 1.  Tipo de provedor: preencha o tipo de provedor de acordo com as categorias em questão 7, onde o paciente procurou tratamento ou orientação.  Tempo de viagem: horas gastas para viajar de e para a instalação  Tempo gasto para a visita: preencha o horário para visitas ambulatoriais e hospitalizações  Encargos do dia: taxas para os dias do hospital. Apenas para hospitalizações, e apenas para ser preenchido se não for coberto pelos itens de custo abaixo (taxa de consulta, radiografia etc.)  Taxa de consulta: outros encargos, não cobertos pela taxa diária, incluindo pagamento direto ao pessoal de saúde  Radiografia e outras imagens: pagamentos para investigação de imagem (raios-x, tomografia computadorizada, ultra-som). Específicos para TB e outros  Taxas de teste de laboratório: pagamentos para todos os testes, específicos para TB e outros  Outros procedimentos: pagamentos para biópsia, lavagem brônquica, etc., mas não cirurgia não relacionada à TB  Taxas de medicamentos: Qualquer medicamento (TB ou outro) prescrito antes de ser diagnosticado com tuberculose pelo NTP  De outros. incluindo suplementos nutricionais: quaisquer outros tratamentos, tais como suplementos nutricionais indicados  Viagem: pagamentos para viagens até a instalação (não inclui perda de receita). tanto para o paciente como para qualquer membro do agregado familiar.  Comida: pagamentos diretos por alimentos adicionais comprados em relação à viagem de cuidados de saúde e durante a visita ou hospitalização, tanto para pacientes como para quaisquer membros do agregado familiar  De outros. incluindo alojamento: inclui pagamentos diretos relacionados com o aluguer de um quarto / cama durante as visitas de cuidados de saúde e quaisquer outros pagamentos não médicos relacionados com a visita de cuidados de saúde, tanto para o doente como para qualquer membro do agregado  Reembolso do seguro saúde: o montante reembolsado ao paciente através do seguro médico (privado ou previdenciário) até o momento, não inclui o reembolso futuro esperado  Pagamentos diretos (brutos): pagamento direto aos prestadores de serviços de saúde por indivíduos no momento do uso do serviço, isto é, excluindo pagamento antecipado de serviços de saúde - por exemplo, na forma de impostos ou de prêmios ou contribuições de seguro específicos. calculado como a soma dos custos médicos diretos (A) e diretos não médicos (B). Se o paciente não puder lembrar os detalhes dos custos acima, pedir o total de pagamentos da visita, hospitalização.  Pagamento direto (líquido): pagamentos diretos médicos e não-médicos menos os reembolsos. | |

|  |  |  |  | | Pagamentos médicos do próprio bolso. (Total por visita )  (A) | | | | | | | Pagamentos não-médicos do próprio bolso. (Total por visita)(B) | | | | | Pagamentos do próprio bolso (A+B) | (C) | Próprio bolso pagamentos por estadia (A+B-C) |
| --- | --- | --- | --- | --- | --- | --- | --- | --- | --- | --- | --- | --- | --- | --- | --- | --- | --- | --- | --- |
| Visita # | Semanas antes do início do tratamento | Tipo de provedor (ver lista) | Tempo de viagem (horas): | Tempo gasto para visitar (horas): | Dia (para internações somente) A1 | Taxa de consulta A2 | Radiografia e outras imagens A3 | Testes laboratoriais  A4 | Outros procedimentos  A5 | Medicamentos A6 | Pagamentos de médicos. total ΣA1-6 | Viagens B1 | alimento durante a cuidados de saúde ou hospital ficar  B2 | Alojamento  B3 | Suplementos nutricionais  B4 | Próprio bolso pagamentos não-médicos (Total)  ΣB1-4 | Total dos pagamentos do próprio bolso (ΣA1-7) + (ΣB1-4) | Reembolso de seguro de saúde |  |
| 1^st^ |  |  |  |  |  |  |  |  |  |  |  |  |  |  |  |  |  |  |  |
| 2^nd^ |  |  |  |  |  |  |  |  |  |  |  |  |  |  |  |  |  |  |  |
| 3^rd^ |  |  |  |  |  |  |  |  |  |  |  |  |  |  |  |  |  |  |  |
| 4^th^ |  |  |  |  |  |  |  |  |  |  |  |  |  |  |  |  |  |  |  |
| 5^th^ |  |  |  |  |  |  |  |  |  |  |  |  |  |  |  |  |  |  |  |
| 6^th^ |  |  |  |  |  |  |  |  |  |  |  |  |  |  |  |  |  |  |  |
| 7^th^ |  |  |  |  |  |  |  |  |  |  |  |  |  |  |  |  |  |  |  |

| **Parte III. Custo durante o atual tratamento de TB/MDR-TB (deve ser preenchido para todos os pacientes), a menos que especificado, esta seção refere-se a tratamento somente da fase atual do paciente** | | | | | | | | | | | | | | | | | | | | | | | | |
| --- | --- | --- | --- | --- | --- | --- | --- | --- | --- | --- | --- | --- | --- | --- | --- | --- | --- | --- | --- | --- | --- | --- | --- | --- |
| **Questão** | | | | | | Categorias resposta (marque todas que se aplicam ou preencher a resposta na linha de resposta) | | | | |  | | Instruções e as ações para o entrevistador | | | | | | | | | | | |
| 1. Você atualmente está hospitalizado devido TB? | | | | | | 1. Sim 2. Não | | | | |  | | Se sim, os dados de custo coletados aplica-se a primeira linha da pergunta da tabela 29 | | | | | | | | | | | |
| 1. Você anteriormente foi hospitalizado durante sua fase atual de tratamento de TB e por causa de TB? | | | | | | 1. Sim_____Tempo 2. Não | | | | |  | | Refere-se apenas de hospitalização durante a fase de tratamento atual: não inclui internação antes de começar o tratamento de TB atual: novos casos, internações antes do tratamento de TB ter começado devem ser preenchidas em parte III. Se resposta para ambos pergunta 27 e 28 são "não", Então pule para a pergunta 30. | | | | | | | | | | | |
| 1. Sobre quanto dinheiro e tempo gastou para cada uma dessas internações? | | | | | | • Veja a tabela abaixo e peça cada item. Preencha uma linha por visita.  Explicação dos títulos da tabela:  Tipo de hospital: preencha o tipo de provedor de acordo com as categorias em questão 6  Número de dias hospitalizados: inclui consultas ambulatoriais e hospitalizações. Deve ser preenchido em ordem cronológica  Cobranças diárias: taxas totais para os dias do hospital. Apenas para ser preenchido, se não coberto pelos itens de custo abaixo  Taxa de consulta: outros encargos, não cobertos sob carga do dia, incluindo pagamento direto ao pessoal de saúde  Radiografia e outras imagens: qualquer investigação de imagem (raios-x, tomografia computadorizada, ultra-som), específica para TB e outros  Taxas de exames de laboratório: inclui todos os testes, específicos para TB e outros, incluindo o custo de transporte de amostras, se pagos pelo paciente  Outros procedimentos: inclui biópsia, lavagem brônquica, etc., mas não cirurgia não relacionada à TB  Medicamento para tratar a tuberculose: taxas apenas para medicamentos contra a tuberculose, comprados dentro ou fora do hospital  Outros medicamentos incluindo suplementos nutricionais: qualquer outro medicamento, incluindo suplementos nutricionais  Pagamentos diretos (brutos): É a soma de despesas médicas e não médicas. Se o paciente não se lembrar dos detalhes dos pagamentos acima, ou tiver uma conta hospitalar para todos os custos combinados, peça o pagamento total do desembolso da hospitalização.  Pagamento direto (líquido): soma de pagamentos diretos médicos e não-médicos menos reembolsos.  Viagem: pagamento do próprio bolso para viagem até a instalação (não inclui perda de receita), tanto para o paciente quanto para qualquer membro do domicílio.  Suplementos nutricionais  Comida: pagamento direto de alimentos comprados em relação a viagens para e durante a hospitalização, paciente e membro da família.  Outros, incluindo acomodação: pagamentos relacionados com o aluguel de um quarto / cama durante visitas de cuidados de saúde e quaisquer outras despesas não médicas para o doente e membro do agregado.  Reembolso do seguro de saúde: valor reembolsado ao paciente até o momento, não inclui o reembolso futuro esperado | | | | | | | | | | | | | | | | | | |
|  |  |  | | Pagamentos médicos de próprio bolso. (Total por estadia) (A) | | | | | | | | | | | | Pagamentos não-médicos do próprio bolso. (Total por estadia)  (B) | | | | | | Próprio bolso pagamentos por estadia (A+B) | (C) | Próprio bolso pagamentos por estadia (A+B-C) |
| Hospitalização | Tipo de hospital (ver lista) | Número de dias hospitalizado | Tempo de viagem (horas) | Encargas do dia (totais de estadia) A1 | Taxa de consulta (total por estadia)  A2 | | Radiografia e outras imagens (total por estadia)  A3 | Testes de laboratório, incluindo o custo de transporte de amostras (totais por estadia)  A4 | Outros procedimentos. incluindo a cirurgia. biopsia.etc A5 | | | Outros medicamentos. (total de estadia)  A6 | | Pagamentos médico (Total)  ΣA1-6 | | Viagens (total por estadia)  B1 | | Alimentos (total por estadia)  B2 | Suplementos nutricionais  B3 | Outros (pagamento de linho... sabão. outros serviços e administrativo) (total por estadia)  B4 | Pagamentos de próprio bolso não-médicos (Total)  ΣB1-4 | Total dos pagamentos do próprio bolso | Reembolso de seguro de saúde |  |
| 1^st^ |  |  |  |  |  | |  |  |  | | |  | |  | |  | |  |  |  |  |  |  |  |
| 2^nd^ |  |  |  |  |  | |  |  |  | | |  | |  | |  | |  |  |  |  |  |  |  |
| 3^rd^ |  |  |  |  |  | |  |  |  | | |  | |  | |  | |  |  |  |  |  |  |  |
| 4^th^ |  |  |  |  |  | |  |  |  | | |  | |  | |  | |  |  |  |  |  |  |  |
| 5^th^ |  |  |  |  |  | |  |  |  | | |  | |  | |  | |  |  |  |  |  |  |  |
| 6^th^ |  |  |  |  |  | |  |  |  | | |  | |  | |  | |  |  |  |  |  |  |  |
| **Custos para TDO durante ambulatório** | | | | | | | | | | | | | | | | | | | | | | | | |
| **Questão** | | | | | | | | | | Categorias de respostas (marque todas que se aplicam ou preencher a resposta na linha de resposta) | | | | | | | Ação é o entrevistador | | | | | | | |
| 1. Em uma base diária, atualmente toma seus medicamentos sem supervisão ou apoio (autoadministrada) ou tem um supervisor de tratamento (TDO)? | | | | | | | | | | 1. Auto administrado  2. TDO  3. Auto administrado e TDO | | | | | | | - TDO (tratamento diretamente observado) é para a supervisão da ingestão diária de medicamentos, ou seja, o que é feito todos os dias. - Estas questões não estão se referindo ao menos frequentes viagens para retirar medicamentos (por ex. semanais), que são exploradas de pergunta 38 em diante. Esta pergunta refere-se a fase de tratamento, em que o paciente está atualmente. | | | | | | | |
| 1. Se TDO, quantas vezes por semana? | | | | | | | | | |  | | | | | | | O máximo será 7 vezes por semana | | | | | | | |
| 1. Se você está agora na fase de continuação, tomou seus medicamentos na fase intensiva sem supervisão ou apoio (auto administrada), ou você tem um supervisor de tratamento (TDO)? | | | | | | | | | | 1. Auto administrado  2. TDO em fase intensiva,  ________ tempo por semana  3. TDO em fase de continuação,  ________tempo por semana | | | | | | | O máximo será 7 vezes por semana | | | | | | | |
| 1. Se TDO, quem é o provedor do TDO? | | | | | | | | | | 1. Unidade de saúde  2. Trabalhador de saúde comunitário / voluntário  3. Membro da família  4. Outros | | | | | | |  | | | | | | | |
| 1. Se TDO, quanto tempo gastou na última tomada de visita TODO, incluindo o tempo de viagem e tempo de espera (tempo de retorno total)? | | | | | | | | | | ….. horas | | | | | | |  | | | | | | | |
| 1. Qual foi o custo de transporte (retorno) para a última visita TODO, incluindo os custos de estacionamento, no total para você e qualquer membro do agregado que acompanha? | | | | | | | | | |  | | | | | | |  | | | | | | | |
| 1. Havia alguma taxa paga ao provedor TDO? | | | | | | | | | | 1. Sim  Se sim, quanto:____________  2. Não | | | | | | |  | | | | | | | |
| 1. Quanto gastou com alimentos e bebidas para a última visita TDO (na estrada. enquanto espera... almoço etc.). no total para você e qualquer membro do agregado que acompanha? | | | | | | | | | |  | | | | | | |  | | | | | | | |
| Custos para retirada de medicamentos durante tratamento ambulatorial (devemos acrescentar nota para que a visita TDO e a de retirada de medicamentos não sejam confundido uma com a outra – retirada de medicamentos é muito mais curta do que o TDO e não necessariamente o mesmo tipo de cuidados de saúde e trabalhador de saúde são envolvidos] | | | | | | | | | | | | | | | | | | | | | | | | |
| 1. Você ou um membro do agregado familiar faz retirada de medicamentos para TB (para o tratamento auto administrado ou para trazer para o seu TODO supervisor/suporte)? | | | | | | | | | | 1. Sim. 2. Não | | | | | | | Isso não diz respeito às visitas ao TDO, que devem ser registradas nas perguntas 30-37, mas devem ser preenchidas se o paciente ou outro membro do domicílio pegam medicamentos para levar ao supervisor de TDO ou para tratamento auto administrado.  Se o paciente estiver no TDO e o paciente ou membro da família não estiver pegando drogas para levar ao supervisor do TDO, a resposta é não.  Se não, pule para a questão 46. | | | | | | | |
| 1. Se sim. Quantas vezes que você ou um membro do agregado familiar retira medicamentos para TB na fase atual de tratamento? | | | | | | | | | | 1. Todos os dias  2. Toda semana  3. Duas semanas por mês  Outros___ | | | | | | |  | | | | | | | |
| 1. Onde você ou seu membro do agregado retira seus medicamentos para TB? | | | | | | | | | | 1. Unidade de saúde da família  2. Unidade básica de saúde  3. Unidade de referência para tuberculose  4. Unidade de cuidados de emergência  5. Hospital  6. ONGs  7. Serviço privado  8.Outros, especificar:_________ | | | | | | | Se o paciente tem visitado lugares diferentes, assinale o que de mais recente. | | | | | | | |
| 1. Havia alguma taxa paga para retirada de medicamentos? | | | | | | | | | | 1. Sim  Se sim, quanto:____________  2. Não | | | | | | |  | | | | | | | |
| 1. Que custos de alojamento você e qualquer membro do agregado que o acompanha teve quando foi retirar medicamentos na última vez? | | | | | | | | | |  | | | | | | |  | | | | | | | |
| 1. Quanto tempo a última visita de retirada de medicamentos levou, incluindo o tempo de viagem e tempo de espera (tempo de retorno total)? | | | | | | | | | | _____ horas | | | | | | |  | | | | | | | |
| 1. Qual foi o custo do transporte (retorno) da última vez que você retirou medicamentos, incluindo os custos de estacionamento, no total para você e qualquer membro do agregado familiar que o acompanha? | | | | | | | | | |  | | | | | | |  | | | | | | | |
| 1. Quanto gastou com alimentos e bebidas da última vez que você retirou medicamentos (na estrada. enquanto espera... almoço etc.), no total para você e qualquer membro do agregado que acompanha? | | | | | | | | | |  | | | | | | |  | | | | | | | |
| **Custo durante as visitas para acompanhamento médico ambulatorial (consulta ao médico ou enfermeiro, exames)** | | | | | | | | | | | | | | | | | | | | | | | | |
| **Questão** | | | | | | | | | | Categorias repostas (marque todas as aplicar ou preencher a resposta na linha de resposta) | | | | | Ação para o entrevistador | | | | | | | | | |
| 1. Quantas consultas de acompanhamento médico relacionadas à TB você teve até agora durante esta fase de tratamento (para consultar o médico ou enfermeira, fazer exames de acompanhamento, etc.)? | | | | | | | | | | ____tempo | | | | | Isso se refere a check-up clínico, acompanhamento e visitas adicionais devido a efeitos colaterais ou outros problemas relacionados à tuberculose. Não inclui visitas ao TDO ou visitas para pegar medicamentos.  Para pacientes em fase de continuação, pergunte apenas quantas visitas desde o início da fase intensiva. | | | | | | | | | |
| 1. Quanto tempo levou para viajar para o estabelecimento de saúde (tempo em horas levado de casa para o estabelecimento de saúde) | | | | | | | | | | _____horas | | | | | (tempo em horas levado de casa para o estabelecimento de saúde, há uma maneira) | | | | | | | | | |
| 1. Quanto tempo que você permaneceu na instalação (e esperado) desde o momento em que você chegou? | | | | | | | | | | _____horas | | | | | Tempo em horas de tempo de espera para assistência do pessoal de instalação ao paciente | | | | | | | | | |
| 1. Qual foi o custo de transporte (retorno) para a última visita de acompanhamento médico ambulatorial, incluindo estacionamento, no total para você e qualquer membro do agregado que acompanha? | | | | | | | | | |  | | | | | Custo relacionado com a última visita. Se a entrevista ocorrer no final de uma visita, então use os custos para a presente visita. | | | | | | | | | |
| 1. Qual o custo de acomodação que teve para a última visita, no total, para você e qualquer membro do agregado que acompanha? | | | | | | | | | |  | | | | | Custo relacionado com a última visita. Se a entrevista ocorrer no final de uma visita então use os custos para a presente visita. | | | | | | | | | |
| 1. Quais as taxas que você pagou durante sua última visita de acompanhamento médico ambulatorial para registo/consulta? | | | | | | | | | | Taxas registro/consulta……… | | | | | Custo relacionado com a última visita. Se a entrevista ocorrer no final de uma visita então use os custos para a presente visita. | | | | | | | | | |
| 1. Quais as taxas que você pagou durante sua última visita de acompanhamento médico ambulatorial para radiografia e outras imagens? | | | | | | | | | |  | | | | | Consulte a tabela acima para explicações | | | | | | | | | |
| 1. Quais as taxas que você pagou durante sua última visita de acompanhamento médico ambulatorial para testes, testes de TB e outros? | | | | | | | | | | Taxas para exames ……… | | | | | Custo relacionado com a última visita. Se a entrevista ocorrer no final de uma visita então use os custos para a presente visita. | | | | | | | | | |
| 1. Quais as taxas que você pagou durante sua última visita de acompanhamento ambulatorial médica para outros procedimentos? | | | | | | | | | |  | | | | | Custo relacionado com a última visita. Se a entrevista ocorrer no final de uma visita então use os custos para a presente visita. | | | | | | | | | |
| 1. Quais as taxas que você pagou na sua última visita de acompanhamento ambulatório médico para medicamentos TB, incluindo prescrições de medicamentos comprados fora da instalação? | | | | | | | | | | Taxas de drogas ………. | | | | | Custo relacionado com a última visita. Se a entrevista ocorrer no final de uma visita então use os custos para a presente visita. | | | | | | | | | |
| 1. Quais as taxas que você pagou durante sua última visita de acompanhamento ambulatorial médica para outros medicamentos, incluindo os suplementos nutricionais? | | | | | | | | | |  | | | | | Custo relacionado com a última visita. Se a entrevista ocorrer no final de uma visita então use os custos para a presente visita. | | | | | | | | | |
| 1. Que outras taxas não constantes as perguntas anteriores você pagou durante sua última visita de acompanhamento ambulatorial médica? | | | | | | | | | | Outras taxas …………. | | | | | Custo relacionado com a última visita. Se a entrevista ocorrer no final de uma visita então use os custos para a presente visita. | | | | | | | | | |

| **Custos para suplementos nutricionais/alimentares** | | | | |
| --- | --- | --- | --- | --- |
| 1. Você compra/comprou qualquer suplementos nutricionais fora de sua dieta regular devido a doença TB, por exemplo vitaminas recomendadas pela equipe de cuidados de saúde? | 1. Sim 2. Não | | | Se não, pule para a questão 60 |
| 1. Se sim, quanto gastou com estes suplementos nutricionais na semana passada aproximadamente? |  | | |  |
| 1. Você compra/comprou qualquer alimento adicional fora de sua dieta regular devido a doença TB, por exemplo carne. bebidas energéticas. ou frutas, conforme recomendado pela equipe de cuidados de saúde? | 1. Sim 2. Não | | | Se não, pule para a pergunta 62 |
| 1. Se sim, quanto gastou com essa comida adicional na semana passada aproximadamente? |  | | |  |
| - **Perda de tempo para encarregados de educação não deve ser preenchida se o paciente é inferior a 15 anos-para crianças. todas as questões relativas a custos, tempo gasto, renda, e a perda de rendimento nas secções II e o custo de preocupação III para o guardião. Nota: despesas de transporte, comida, alojamento para guardião devem ser incluídas em perguntas na parte III (tabelas).** | | | | |
| **Questão** | | **Categorias de respostas** | Ação para o entrevistador | |
| 1. Alguém da sua família te acompanha para última visita: 2. Visita TDO 3. Visite para buscar medicamentos (ou buscar medicamentos para você) 4. Acompanhamento de visita médica 5. Hospitalização | | 1. Sim 2. Não  1. Sim 2. Não  1. Sim 2. Não  1. Sim 2. Não | Várias respostas possíveis, perda de tempo deve ser calculado com respostas anteriores pelo paciente | |
| 1. Se sim (para qualquer), essa pessoa perdeu uma renda durante esse tempo? | | 1. Sim 2. Não | Se várias respostas em questão 62, perguntar sobre a mais recente visite que um membro do agregado familiar acompanhou. | |

| **Regime de seguro de saúde** | | |
| --- | --- | --- |
| **Questão** | Categorias de respostas (marque todas que se aplicam ou preencher a resposta na linha de resposta) | Ação para o entrevistador |
| 1. Tem qualquer um dos seguintes tipos de seguro de saúde (plano de saúde)? | ___ Nenhum  ___Regime de reembolso ___Coparticipação ou parcial cobertura  ___Total cobertura  ___Outro especifique "Outro". _ |  |

| **Posição social** | | | | |
| --- | --- | --- | --- | --- |
| **Questão** | | Categorias de respostas (marque todas que se aplicam ou preencher a resposta na linha de resposta) | Ação para o entrevistador se paciente estiver abaixo dos 15 anos de idade, estas perguntas dizem respeito a guardião | |
| 1. Qual é seu nível de escolaridade (do paciente)? | | 1. Analfabeto / Fundamental Incompleto  2. Fundamental I completo / Fundamental II incompleto  3. Fundamental II completo / Médio incompleto  4. Médio Completo / Incompleto Superior  5. Completo Superior | Se o paciente está abaixo dos 15 anos, esta pergunta é para o guardião. | |
| 1. Qual é a sua ocupação principal? | | 1. _Estudante  2. _Trabalhador  3. _Agricultor  4. _Aposentado  5. _Doméstico/Casa  6. _Desempregado  7. _Outros__________________________ | Se o paciente é inferior a 15 anos, esta pergunta é para o guardião. | |
| 1. Qual foi o seu emprego principal ou trabalho formal, ou outra atividade principal antes de você contrair TB? | | 1.__Nenhum  2.___ Empregado  3.___ Trabalho doméstico  4.___ Militares de exército, marinha, aeronáutica, polícia militar ou fogo militar  5.___ Emprego no setor privado  6.___ Emprego no setor público  7.___ Empregador  8.___ Conta própria  9.___ Trabalhador desempregado, ajuda do membro do agregado familiar ou pai  10. ___ Estudante  11.___ Outros  Especifique outro". | Se o paciente está abaixo dos 15 anos, esta pergunta é para o guardião. Isto refere-se a tempo antes de desenvolveram sintomas de TB. Todas as opções o nome primeiro | |
| 1. Qual é o seu emprego principal? ou trabalho formal, ou outra atividade principal agora? | | 1.___ Nenhum  2.___ Empregado  3.___ Trabalho doméstico  4.___ Militares de exército, marinha, aeronáutica, polícia militar ou fogo militar  5.___ Emprego no setor privado  6.___ Emprego no setor público  7.___ Empregador  8.___ Conta própria  9.___ Trabalhador desempregado, ajuda do membro do agregado familiar ou pai  10. ___ Estudante  11.___ Outros  Especifique outro". | Se o paciente tiver menos de 15 anos, essa pergunta é para o guardião.  Isso se refere ao tempo antes do desenvolvimento dos sintomas da TB. Nomeie todas as opções primeiro | |
| **Medir a capacidade do agregado familiar de pagar pela saúde usando três métodos: 1) Despesa de consumo (medida 1); 2) Ativos domésticos e nível de educação (medida 2) e renda autodeclarada (medida 3)**  **A renda autodeclarada (medida 3) é a medida menos preferida em países como o Brasil, com economia informal substancial. A integralidade dos “bens domésticos” (medida 2) e “despesas de consumo doméstico” (medida 1) é, portanto, fundamental para estimar a renda ou os gastos das famílias. O estudo irá comparar os resultados usando qualquer uma dessas três medidas. Essas perguntas permitirão estimar a capacidade da família de pagar pelo denominador da medida de custo catastrófico, bem como medir os custos indiretos**. | | | | |
| **Ativos domésticos (medida 2)**  **As perguntas estão usando as definições do IBGE e da ABEP baseadas nos Critérios de Classificação Econômica do Brasil (CCEB). É um sistema de pontuação em que as famílias são classificadas em cinco categorias (quintis). Em 2016, os quintis variaram de menos de R $ 1874 para acima de R $ 18740. As perguntas abaixo são combinadas com perguntas sobre o nível de educação para ajudar a estimar a renda de um lar**. | | | | |
| 1. Sua casa tem? (Marque com X) | | \|  \| Sim \| Não \| \| --- \| --- \| --- \| \| 1. Instalação sanitária \|  \|  \| \| 1. Veículo com quatro rodas \|  \|  \| \| 1. Computador \|  \|  \| \| 1. Lava-louças \|  \|  \| \| 1. Geladeira \|  \|  \| \| 1. Congelador \|  \|  \| \| 1. Máquina de lavar \|  \|  \| \| 1. DVD \|  \|  \| \| 1. Microondas \|  \|  \| \| 1. Moto \|  \|  \| \| 1. Máquina de secar roupa \|  \|  \| |  | |
| 1. O seu agregado familiar emprega trabalhadores domésticos? | | 1. Sim  2. Não |  | |
| 1. O seu agregado familiar tem acesso a água canalizada? | | 1. Sim  2. Não |  | |
| 1. Sua casa está em uma rua pavimentada? | | 1. Sim  2. Não |  | |
| **Despesa de consumo das famílias (Medida 1)**  **Essas perguntas permitem estimar a capacidade de pagamento da família. Perguntas foram extraídas de “Pesquisa de Orçamentos Familiares 2017-2018”**  Se o paciente estiver atualmente residindo em família diferente (com parentes ou amigos), peça ao paciente para pensar em uma semana/mês enquanto residia no domicílio original (a família a qual o paciente pertence). | | | | |
| **DESPESAS SEMANAIS - 7 DIAS** |  | |  |  |
| 1. Por favor, dê a sua despesa semanal em alguns itens como alimentos e bebidas, higiene pessoal e produtos de limpeza doméstica, combustíveis domésticos (exceto gás e lenha), alimentos e suprimentos para animais, outras pequenas compras (vela, pilha, lâmpada, etc.) itens, jogos e apostas, transporte TODAS AS SEMANAS | Alimentos e bebidas  ___________________  Outros produtos  ____________________ | |  |  |
| **DESPESAS TRIMESTRAIS - 90 DIAS** |  | |  |  |
| 1. Por favor, dê a sua despesa mensal em alguns itens como utilitários, combustível doméstico e outros POR TRIMESTRE (90 DIAS)? | 1. energia elétrica _____________  2. água e esgoto ______________  3. Combustível de automóvel ______________  4. telefone residencial (despesa total) __________  5. telefone fixo, celular e internet (pacote) _______  6. telefone fixo e internet (pacote) _____________  7. Acesso à internet (provedor, cabo, comunicação via satélite, etc.) ______________  8. TV paga (pacote) ______________ | |  |  |
| 1. Por favor, dê sua despesa mensal em alguns itens, como combustíveis domésticos e outros | 1. Botijão de gás _________  2. Água para uso geral _________  3. Querosene _________  4. Álcool _________  5. Lenha _________ | |  |  |
| 1. Conservação, manutenção e pequenas reparações com alojamento, depósito e jardinagem | **_____________________________** | |  |  |
| 1. Reparação e manutenção de mobiliário, equipamento, ferramentas e utensílios para uso doméstico | **_____________________________** | |  |  |
| 1. Comunicações | **_____________________________** | |  |  |
| 1. Transporte coletivo | **_____________________________** | |  |  |
| 1. Comida fora de casa | **_____________________________** | |  |  |
| 1. Aquisição de mercadorias de tabaco | **_____________________________** | |  |  |
| 1. Jogos e apostas | **_____________________________** | |  |  |
| 1. Aquisição de jornais, revistas e passatempos impressos | **_____________________________** | |  |  |
| 1. Cuidados pessoais e outros serviços | **_____________________________** | |  |  |
| 1. Aquisição de papelaria, livros não docentes e assinaturas de periódicos | **_____________________________** | |  |  |
| 1. Aquisição de brinquedos e material de lazer | **_____________________________** | |  |  |
| 1. Aquisição e aluguel de roupas (mulheres e homens) | **_____________________________** | |  |  |
| 1. Aquisição e aluguel de roupas infantis até 14 anos | **_____________________________** | |  |  |
| 1. Aquisição de tecidos para vestuário e roupas de banho, cama e mesa | **_____________________________** | |  |  |
| 1. Aquisição e locação de bolsas, sapatos, cintos e outros acessórios | **_____________________________** | |  |  |
| 1. Aquisição de utensílios diversos de banho e cozinha | **_____________________________** | |  |  |
| 1. Outras aquisições | **_____________________________** | |  |  |
| 1. Banco, tabelião, advogado, corretor e serviços similares | **_____________________________** | |  |  |
| 1. Acessórios e manutenção de veículos | **_____________________________** | |  |  |
| 1. Viagem no período de referência | **_____________________________** | |  |  |
| 1. Além das análises de custos TRIMESTRAIS que você forneceu, quanto você acha que no total gasta a cada TRIMESTRE (90 DIAS)? | **_____________________________** | |  |  |
| **DESPESAS E CONSUMO PESSOAL MENSAL (30 DIAS)** |  | |  |  |
| Além dos custos mensais que você forneceu, quanto no total você acha que gasta todo mês (30 dias)? Aquisição de produtos farmacêuticos, higiene pessoal e maquiagem ou ingressos para eventos esportivos e culturais e uso de telefones celulares TODOS OS MESES? | **TOTAL_____________________________** | |  |  |
| 1. Aquisição de produtos farmacêuticos | **_____________________________** | |  |  |
| 1. Artigos de higiene pessoal e maquiagem | **_____________________________** | |  |  |
| 1. Diversos, ingressos para eventos esportivos e culturais e uso de celulares | **_____________________________** | |  |  |
| **DESPESAS ANUAIS DE DOMICÍLIO E CONSUMO (12 MESES)** |  | |  |  |
| Por favor, dê a sua despesa anual em alguns dos itens, como construção e reformas ou melhorias em habitação (veja a lista abaixo!) TODOS OS ANOS? | **_____________________________** | |  |  |
| 1. Construção e reformas de moradias e sepulturas | **_____________________________** | |  |  |
| 1. Outros itens do domicílio principal com serviços públicos, privados e de habitação | **_____________________________** | |  |  |
| 1. Aluguel de eletrodomésticos e utilidades domésticas (telefone residencial, televisão, dvd, microcomputador) | **_____________________________** | |  |  |
| 1. Aquisição de eletrodomésticos, máquinas e outras utilidades para uso doméstico | **_____________________________** | |  |  |
| 1. Aquisição de ferramentas, animais domésticos, equipamentos musicais e camping | **_____________________________** | |  |  |
| 1. Aquisição de móveis | **_____________________________** | |  |  |
| 1. Aquisição de artigos de decoração e forração | **_____________________________** | |  |  |
| 1. Serviços domésticos | **_____________________________** | |  |  |
| 1. Cerimônias familiares, práticas religiosas, outras celebrações e recepções | **_____________________________** | |  |  |
| 1. Compras de joias, relógios, aparelhos e acessórios para telefonia móvel | **_____________________________** | |  |  |
| 1. Outras propriedades | **_____________________________** | |  |  |
| 1. Contribuições, transferências e encargos financeiros | **_____________________________** | |  |  |
| 1. Cursos, livros didáticos, revistas técnicas e outros itens relacionados à educação | **_____________________________** | |  |  |
| 1. Veículos: documentação, seguros e outros | **_____________________________** | |  |  |
| 1. Outras despesas | _____________________(despesa anual total)_________________________ | |  |  |
| 1. Aproximadamente quanto você gasta por ano em itens como educação, vestuário, calçados, capital etc.)? | ________ (Despesa total no mês passado (Real)_____________________ | |  |  |

| Rendimento autodeclarado antes de contrair TB  Essas perguntas permitirão estimar a capacidade de pagamento da família - Medida 3. Em um país como o Brasil com tamanho substancial de trabalho informal, as respostas a essas perguntas serão examinadas criticamente e comparadas com a renda estimada com base nas despesas de consumo (ou seja, “Capacidade de pagamento - medida 1”) e propriedade de bens com nível de escolaridade (Critérios de classificação econômica do Brasil; “Capacidade de pagamento - medida 2”) | | | | | | |
| --- | --- | --- | --- | --- | --- | --- |
| 1. Você foi a pessoa que obteve a maior renda em sua casa antes de contrair TB? | 1. Sim 2. Não, outra pessoa no agregado familiar tinha um rendimento mais elevado 3. Não, a renda era semelhante a outros membros da família | | | Se o paciente tiver menos de 18 anos, essa pergunta é para o responsável. | | |
| 1. Quantas horas por semana você estava trabalhando antes de contrair TB? |  | | | Se o paciente tem menos de 15 anos, essa pergunta é para o guardião.  Isso se refere ao tempo antes do desenvolvimento dos sintomas de TB. | | |
| 1. Se você estava em um trabalho remunerado, quanto você estima que foi o seu rendimento líquido de atividades relacionadas ao trabalho, por mês antes de você contratar TB? |  | | | Se o paciente tem menos de 15 anos, essa pergunta é para o guardião.  Para trabalhadores sazonais que experimentam salários flutuantes, tente determinar uma renda mensal média para essa questão. | | |
| 1. Quanto você estima o rendimento líquido de atividades relacionadas ao trabalho de sua residência por mês, antes de contrair tuberculose? (A renda de todos os membros da família deve ser contada) |  | | | Refere-se a todas as pessoas da casa.  Para trabalhadores sazonais que experimentam salários flutuantes, tente determinar uma renda mensal média para essa questão. | | |
| **Mudanças de renda e consequências sociais** | | | | | | |
| **Questão** | | **Categorias de respostas** (circule o mais adequado ou preencha a resposta na linha de resposta) | | | | **Ação para o entrevistador**  Se o paciente tem menos de 15 anos de idade, essas questões dizem respeito ao responsável |
| 1. Durante a fase intensiva, você teve que mudar de emprego ou profissão ou parou de trabalhar? | | 1. Sim 2. Não | | | |  |
| 1. Durante a fase de continuação, você teve que mudar de emprego ou profissão ou parou de trabalhar? | | 1. Sim 2. Não | | | |  |
| 1. Se você está em um trabalho remunerado, o quanto você estima o lucro líquido de atividades relacionadas ao trabalho. por mês é agora? | |  | | | | Se o paciente tem menos de 15 anos, essa pergunta é para o guardião.  Ao se estabelecer em um setor informal importante, você pode não querer se referir explicitamente a impostos para garantir que as pessoas estejam respondendo corretamente. |
| 1. Quanto você estima que foi o rendimento líquido de atividades relacionadas ao trabalho de sua casa por mês, no momento do seu diagnóstico? | |  | | | | Refere-se a todas as pessoas da casa.  Para trabalhadores sazonais que experimentam salários flutuantes, tente determinar uma renda mensal média para essa questão. |
| 1. Quanto você estima o rendimento líquido de atividades relacionadas ao trabalho de sua casa por mês agora? | |  | | | | Refere-se a todas as pessoas no domicílio  Para trabalhadores sazonais que experimentam salários flutuantes, tente determinar uma renda mensal média para essa questão. |
| 1. Quantas horas por semana você está trabalhando agora? | |  | | | | Se o paciente tiver menos de 15 anos, esta questão é para o guardião. |
| 1. Aproximadamente quantos dias úteis de renda você perdeu devido à sua doença de TB em geral? | |  | | | | Dias úteis de rendimento: por exemplo, se um paciente não puder trabalhar por 5 dias e perder renda para eles. O número de dias perdidos é 0,5 * 5 = 2,5. Relatório para episódio total de TB. incluindo todos os dias antes e depois da perda de emprego. |
| 1. Teve que pagar pelo trabalho perdidos (escreva "sim" ou "não") | | **Antes do diagnóstico**   1. Sim 2. Não | | | | **Agora**   1. Sim 2. Não |
| 1. Quantas horas por semana você trabalhou/trabalha? | | **Antes do diagnóstico** | | | | **Agora** |
| 1. Renda por mês (R$) | | **Antes do diagnóstico** | | | | **Agora** |
| 1. Quanto eles foram capazes de manter do total (paciente) salário   How much they were able to keep from the total (patient) salary | | **Antes do diagnóstico** | | | | **Agora** |
| 1. Quanto eles foram capazes de manter do salário doméstico (total)   How much they were able to keep from the household salary (total) | | **Antes do diagnóstico** | | | | **Agora** |
| 1. Você ou sua família receberam algum pagamento de assistência social depois de ter sido diagnosticado com TB? Se sim. Que tipo e quantia durante o último mês? | | 1. Não 2. Auxílio doença ____ por mês. 3. Benefício básico do bolsa família____ por mês. 4. Benefício variável____ por mês. 5. Benefício variável de 0 a 15 anos do bolsa família____ por mês. 6. Benefício variável à gestante do bolsa família____ por mês. 7. Benefício variável à nutriz do bolsa família____ por mês. 8. Benefício variável ao jovem do bolsa família____ por mês. 9. Benefício para superação da extrema pobreza do bolsa família____ por mês. 10. Benefício de Prestação Continuada BPC ____ por mês. 11. Reembolso de seguro de saúde privado ____ por mês. 12. Licença médica remunerada____ por mês. 13. Benefício por invalidez____ por mês. 14. Outro _________, ____ por mês. | | | | Se o paciente tem menos de 15 anos, essa pergunta é para o guardião.  Categorias de acordo com as seguintes categorias  Ao se estabelecer em um setor informal importante, você pode não querer se referir explicitamente a impostos para garantir que as pessoas estejam respondendo corretamente. |
| 1. Você atualmente recebe vales ou bens em espécie para lidar com a doença da tuberculose? | | 1. Sim  a. Vale de transporte: ____por mês  b. Cesta de alimentos: ____por mês  c. Outros. ___por mês  2. Não | | | | Se o paciente tem menos de 15 anos, essa pergunta é para o guardião.  Mais de uma categoria permitida.  Se não, pule para a questão 135 |
| 1. De quem você recebe o vale / bens | | 1. Governo  2. ONGs  3. Empregador  4. Doação privada  5. Outro | | | | Se o paciente tem menos de 15 anos, essa pergunta é para o guardião.  Mais de uma resposta permitida |
| 1. Quantos adultos e crianças dormem regularmente em sua casa? (incluindo paciente, se variável, usar no momento do diagnóstico) | | a. ____ adultos  b. _____ crianças | | | |  |
| 1. Quantos cômodos existem na casa, excluindo o banheiro? | |  | | | |  |
| 1. A doença da tuberculose afetou sua vida social ou privada de alguma forma? | | 1. Não  2. Insegurança alimentar  3. Divórcio ou separado do cônjuge / parceiro  4. Perda de emprego  5. Escolaridade interrompida  6. Exclusão social  7. Outro | | | | Mais de uma categoria permitida. |
| **Enfrentamento** | | | | | | |
| **Questão** | | | **Categorias de respostas** (circule o mais adequado ou preencha a resposta na linha de resposta) | | **Ação para o entrevistador**  Se o paciente tiver menos de 15 anos, estas perguntas são para o guardião. | |
| 1. Você pegou emprestado ou recebeu algum dinheiro para cobrir os custos incorridos desde que iniciou o tratamento para TB? | | | 1. Sim 2. Não | | Se não, vá para a questão 142 | |
| 1. Se sim, quanto você pediu / recebeu (no total)? | | | _____ | |  | |
| 1. De quem você pediu / recebeu? | | | ___ Família / parente próximo  ___ Parente distante  ___ Vizinhos / amigos  ___ Organização diferente de financiamento bancário  ___ Loja de penhores  ___ Empregador  ___ Banco  ___ "Credor não oficial" (agiota)  ___ Outras  Especifique outro | | Múltiplas respostas permitidas. | |
| 1. Você teve/terá que pagar a quantia de volta? | | | 1. Sim 2. Não | |  | |
| 1. Você já vendeu alguma propriedade para financiar o custo incorrido durante o tratamento da TB? | | | 1. Sim 2. Não | | Se não, pule para a questão 145 | |
| 1. Se sim, o que você vendeu? | | | 1. Terra  2. Pecuária  3. Transporte / veículo  4. Item doméstico  5. Produção Agrícola  6. Ouro / joias  7. Outros | | Múltiplas respostas permitidas. Circule tudo o que é mencionado | |
| 1. Quanto dinheiro você recebeu com a venda de todos os itens de sua propriedade (no total)? | | |  | |  | |
| 1. O impacto na sua casa financeiramente desde que você teve sintomas de tuberculose foi que sua família se tornou: | | | 1 = Mais rico  2 = Inalterado  3 = Mais pobre  4 = Muito mais pobre | |  | |

| **Obrigado pela sua colaboração! Existe alguma coisa que você gostaria de perguntar ou dizer?** | | | |
| --- | --- | --- | --- |
|  | | | |
| **Comentários do entrevistador:** | | | |
|  | | | |
| Data (Dia/mês/ano) | ……/……/……. | **Assinatura do entrevistador:** | …………………………………………… |
