# Supplementary material for: The economic burden of households affected by tuberculosis in Brazil: First national survey results, 2019-2021
Source: PLoS One. 2023 Dec 13;18(12):e0287961. doi: 10.1371/journal.pone.0287961 (PMC10718450; doi:10.1371/journal.pone.0287961)
Supplement: S2 File — (PDF) [file pone.0287961.s002.pdf]

9. FORMULÁRIO

ANEXO G	PACIENTE TB CUSTOS - FORMULÁRIO DE PESQUISA PARA O BRASIL	
**PARTE I. INFORMAÇÕES DO PACIENTE OBTIDOS DO CARTÃO DE TRATAMENTO TB ANTES DA ENTREVISTA**		
**QUESTÃO**	**CATEGORIAS DE RESPOSTAS**	**ORIENTAÇÃO PARA O ENTREVISTADOR**
**Selecione o número apropriado ou preencha a linha de resposta. Obs: as perguntas na parte 1 deste formulário, preferencialmente, deverão ser preenchidas com informações do prontuário após assinatura do Termo de Consentimento Livre e Esclarecido (TCLE).**		
**1. Data da entrevista**	(Dia/mês/ano)_____/____/_____	1. Preencher a data seguindo a instrução dd/mm/aaaa
**2. Telefone do participante**		2. Preencha o telefone de contato do participante com o DDD
**3. Selecione um município**		Selecionar o município.
**4. Selecione Região**	1. CENTRO OESTE (CO)<br>2. NORDESTE (NE)<br>3. NORTE (N)<br>4. SUDESTE (SE)<br>5. SUL (S)	Selecionar a região do país onde se localiza o município.
**5. Selecione o local da entrevista (nome do Serviço de Saúde)**	Disponível no ONA (147 Serviços de saúde)<br>Ou preencher, caso o estabelecimento não esteja listado.	Selecionar o nome do Serviço de Saúde

**6. Paciente ID**	X-YYYYYYY-00 (Inserir tabela municípios)	Para a identificação do paciente será utilizado um código com números. Correspondente a região do Brasil (1 - CO, 2 - NE, 3. N, 4. SE, 5. S); município (Código do IBGE) e número do participante no estudo (Número sequencial). Ex: X-YYYYYYY-00 (5-2700300-01) = Nordeste-Arapiraca-01
**7. Nome do entrevistador**	Disponível no ONA	Selecionar o nome do entrevistador cadastrado no ONA.
**8. Tipo de Serviço de Saúde**	1. Atenção Primária<br>2. Especializada	Selecionar o tipo de Serviço de Saúde, se integrante da "Atenção primária" ou integrante da "Atenção Especializada".Atenção Básica é o primeiro nível de atenção em saúde e se caracteriza por um conjunto de ações de saúde, no âmbito individual e coletivo, que abrange a promoção e a proteção da saúde, a prevenção de agravos, o diagnóstico, o tratamento, a reabilitação, a redução de danos e a manutenção da saúde com o objetivo de desenvolver uma atenção integral que impacte positivamente na situação de saúde das coletividades. Este trabalho é realizado nas Unidades Básicas de Saúde (UBS), Unidade de Saúde da Família (USF), Consultório de Rua. Atenção Especializada é uma unidade fragmentada de atendimento que possui uma vasta gama de áreas de diferenciação médica. Ex: Unidades hospitalares, Serviços de Emergência e Serviços ou ambulatórios de referência.

**9. Categoria do Serviço de Saúde de tratamento**	1. Unidade de Saúde da Família (USF)<br>2. Unidade de Saúde da Família (USF) com PCT<br>3. Unidade Básica de Saúde (UBS)<br>4. Unidade Básica de Saúde (UBS) com PCT<br>5. Unidade de Referência para Tuberculose<br>6. Unidade de Urgência e Emergência (Pronto Socorro - PS, Unidades de Pronto atendimento - PA)<br>7. Hospital Público<br>8. Serviço Privado de saúde<br>9. Outro	O serviço de saúde ou "tratamento" é o local onde o paciente realiza o tratamento. Observar a diferenciação em Serviços de Saúde da Atenção Primária (USF, UBS com ou sem PCT) e Serviços Especializados (PAs, Ponto Socorro, Hospital Público), outro, especificar.
**10. Sexo**	1. Masculino<br>2. Feminino	1. Masculino<br>2. Feminino
**11. Data de Nascimento**	(Dia/mês/ano)_____/____/_____ (abrir calendário para registro da data de nascimento)	Preencher a data seguindo a instrução dd/mm/aaaa.
**12. Idade**		Preencher Idade
**13. Data do diagnóstico**	(Dia/mês/ano)_____/____/_____	Preencher a data seguindo a instrução dd/mm/aaaa

**14. Local do diagnóstico**	1. Unidade de Saúde da Família (USF)<br>2. Unidade de Saúde da Família (USF) com PCT<br>3. Unidade Básica de Saúde (UBS)<br>4. Unidade Básica de Saúde (UBS) com PCT<br>5. Unidade de Referência para Tuberculose<br>6. Unidade de Urgência e Emergência (Pronto Socorro - PS, Unidades de Pronto atendimento - PA)<br>7. Hospital Público<br>8. Serviço Privado de saúde<br>9. Outro (Se não tiver no prontuário)	Observar a diferenciação em serviços de saúde da Atenção Primária (USF, UBS com ou sem PCT) e Serviços Especializados (PAs, Ponto Socorro, Hospital Público). Outro, especificar.
**15. Tipo de TB**	1. Pulmonar<br>2. Extrapulmonar<br>3. Pulmonar + Extrapulmonar	Forma clínica da tuberculose, por ocasião da notificação, segundo a sua localização.
**16. Em tratamento de TBDR**	1. Sim<br>2. Não	TBDR, caso de tuberculose em que se verifica resistência in vitro a pelo menos Rifampicina (RMP) e Isoniazida (INH), principais medicamentos utilizados no tratamento da tuberculose.

<table>
<tr><td>17. Tipo de Tratamento</td><td colspan="2">17. A) NÃO TBDR - Esquema Básico 1. Caso novo 2. Recidiva 3. Reingresso após abandono

17. B) TBDR - 4. Caso novo 5. Recidiva 6. Retratamento após abandono 7. Falência ao primeiro tratamento 8. Falência ao retratamento 9. Outros

1. CASO NOVO– Qualquer pessoa que nunca fez tratamento para TB ou o fez por menos de 30 dias. (Esquema Básico – RHZE)
2. RECIDIVA– Caso de TB sensível tratado anteriormente com esquema básico e que recebeu alta por cura comprovada ou por ter completado o tratamento.
3. REINGRESSO APÓS ABANDONO – Caso de TB sensível, mas que deixou de tomar os medicamentos por 30 dias consecutivos ou mais.
4. CASO NOVO– Qualquer pessoa que nunca se submeteu ao tratamento de TBDR, ou o fez por até 30 dias.
5. RECIDIVA – Caso de TBDR tratado anteriormente com esquema para TBDR e que recebeu alta por cura comprovada ou por ter completado o tratamento,
6. REINGRESSO APÓS ABANDONO – caso de TBDR tratado anteriormente com esquema para TBDR, mas que deixou de tomar os medicamentos por 30 dias consecutivos ou mais.
7. FALÊNCIA AO PRIMEIRO TRATAMENTO – caso de TBDR que apresentou falência ao primeiro tratamento de TBDR.
8. FALÊNCIA AO RETRATAMENTO – caso de TBDR que apresentou falência ao retratamento de TBDR.
9. SE OUTROS, especificar.</td></tr>
<tr><td>18. Realizou tratamento anterior para TB</td><td>1. Sim
2. Não</td><td>Algum tratamento anterior ao atual realizado.</td></tr>
<tr><td>19. Duração da fase intensiva do tratamento atual</td><td>_____ meses</td><td>Registrar em meses o tempo planejado da fase intensiva de tratamento. A fase intensiva, ou fase inicial do tratamento é o período de tempo que tem por objetivo reduzir rapidamente a população bacilar e a eliminação de bacilos com resistência natural a algum medicamento, e como consequência</td></tr>
</table>

		a redução da contagiosidade.
**20. Duração total da fase de manutenção do tratamento atual**	_____ meses	Registrar em meses o tempo planejado da fase de manutenção do tratamento.<br>A fase de manutenção, após a fase inicial ou intensiva é o período de tempo que tem por objetivo eliminar os bacilos latentes ou persistentes e reduzir a possibilidade de recidiva da doença.
**21. Data de início do atual tratamento de TB**	(Dia/mês/ano)_____/____/_____	Preencher a data seguindo a instrução dd/mm/aaaa.
**22. O paciente está atualmente em fase de tratamento intensivo ou de manutenção?**	1. Fase intensiva<br>2. Fase de manutenção	O paciente deve ter no mínimo **14 dias de tratamento** na fase intensiva de tratamento, caso contrário deverá ser entrevistado posteriormente a esse período ou ser excluído do estudo.<br>Fase intensiva para regimes de TBDR é o período de tratamento inicial, que inclui, geralmente, uma droga injetável (geralmente 8 meses). A fase de manutenção é o período após a fase inicial ou intensiva concluída e prolonga-se até o final do tratamento.
**23. Quantos dias de tratamento foram concluídos na fase atual?**	______________dias.	O número de dias da fase atual de tratamento será calculado baseado na data de início de tratamento.<br>(abrir calendário para seleção da data)<br>(selecionar a data que está ocorrendo a entrevista)

**24. Status de HIV (conforme indicado no cartão de tratamento)**	1. Positivo<br>2. Negativo<br>3. Não testado/desconhecido	Resultado da sorologia para o vírus da imunodeficiência adquirida (HIV) ou teste rápido, como recomendado pelo MS, realizado anterior ou posteriormente a Notificação de TB.
**25. Doenças associadas**	1. Diabetes<br>2. Hepatite<br>3. Doença Renal Crônica<br>4. Nenhuma<br>5. Outros	Registrar de acordo com as informações do paciente no prontuário ou informações fornecidas pelo responsável do paciente.
**26. Raça/cor**	1. Branca<br>2. Preta<br>3. Amarela<br>4. Parda<br>5. Indígena<br>6. Não declarada	Registrar de acordo com as informações do paciente no prontuário ou informações fornecidas pelo paciente ou responsável do paciente.
**27. Estado Civil**	1. Casado<br>2. Solteiro<br>3. Viúvo<br>4. União estável<br>5. Divorciado/desquitado/separado judicialmente	Registrar de acordo com as informações do paciente no prontuário ou informações fornecidas pelo paciente ou responsável do paciente.
**28. Religião**	1. Católico<br>2. Protestante<br>3. Espírita<br>4. Sem religião<br>5. Outros especificar	Registrar de acordo com as informações do paciente no prontuário ou informações fornecidas pelo paciente ou responsável do paciente.

PARTE II - CAPTA DADOS SOBRE CUSTOS ANTES DO ATUAL TRATAMENTO DE TB (PREENCHIDO APENAS PARA OS CASOS EM FASE INTENSIVA DE TRATAMENTO) DESPESAS DO PRÓPRIO BOLSO, REEMBOLSOS E PERDA DE TEMPO, ANTES E DURANTE O DIAGNÓSTICO DE TB (ANTES DO INÍCIO DO TRATAMENTO DE TB)		
**QUESTÃO**	**CATEGORIAS DE RESPOSTAS**	**ORIENTAÇÃO PARA O ENTREVISTADOR**
**29. Para este episódio de TB, quantas semanas antes de começar o tratamento você experimentou sintomas de TB?**	________Semanas antes do início do tratamento	Registrar o número de semanas antes do início do tratamento. Muitas vezes há um problema que define os “sintomas da tuberculose”, pois os pacientes não sabem que a tosse, a febre ou a perda de peso que inicia o processo de procura de cuidados tem alguma relação com a tuberculose. Portanto, certifique-se de começar com perguntas abertas sobre os sintomas que elas experimentaram nos estágios iniciais da doença e depois perguntar quando esses sintomas ocorreram pela primeira vez, quando pioraram e começaram a preocupar o paciente e levaram o paciente a procurar atendimento. Construa uma linha do tempo de eventos, seja a partir dos primeiros sintomas, ou comece com o tempo de diagnóstico de TB e trabalhe de trás para frente. Se necessário, use um calendário adaptado localmente com os principais eventos sazonais com os quais o paciente pode se relacionar e usar como ponto de referência para o tempo. Use esta linha do tempo para mapear as datas de todos os episódios de busca de cuidados ou conselhos para ajudar a registrá-las nas respostas para a próxima pergunta (ver Pergunta 30). Você só deve investigar os sintomas clássicos de tuberculose da tosse, perda de peso, dor torácica e sudorese noturna e seu tempo se eles não foram relatados durante o questionamento aberto.

30. Quanto dinheiro e tempo gastaram para cada uma destas visitas antes do diagnóstico da tuberculose, incluindo a visita quando você realmente recebeu seu diagnóstico?

VEJA A TABELA ABAIXO E PERGUNTE POR CADA ITEM

1º Preencha uma linha por visita // **2º** Adicione mais linhas se mais visitas forem feitas antes do diagnóstico de TB // **3º** Para todos os que não se aplicam, marque/selecione *NA*

EXPLICAÇÃO DOS TÍTULOS DA TABELA:

VISITAS: inclui visitas ambulatoriais e hospitalizações. Deve ser preenchido em ordem cronológica, primeira visita = VISITA 1.

TIPO DE PROVEDOR: preencha o tipo de provedor com o quadro abaixo:

✓ 1. Unidade de Saúde da Família Unidade	✓ 2. Básica de Saúde / Centro de Saúde
✓ 3. Hospital Público	✓ 4. Hospital Provado ou filantrópico
✓ 5. Clinica Privada	✓ 6. Clínica Popular
✓ **7.** Pronto Socorro Especializado	✓ 8. Unidade de Referência para TB
✓ 9. Programa de Controle da Tuberculose	✓ 10. Policlínica
✓ 11. Curandeiro / Benzedeira	✓ 12. Farmácia ou drogaria
✓ 13. Práticas Integrativas e Complementares	✓ 14. Outro? Especificar.

PAGAMENTOS MÉDICOS

**TEMPO DE VIAGEM**	Horas gastas para viajar até a unidade de saúde
**TEMPO GASTO PARA A VISITA EM HORAS**	Preencha o tempo gasto em horas durante as visitas ambulatoriais (incluindo tempo de espera) e hospitalizações (se esse for o caso multiplicar o número de dias de

		hospitalização por 24 horas).
	**ENCARGOS DO DIA/DIÁRIA**	Apenas para hospitalizações pagas (particular).
	**TAXA DE CONSULTA**	Outros encargos, não cobertos pela taxa diária, incluindo pagamento direto ao pessoal de saúde.
	**RADIOGRAFIA E OUTRAS IMAGENS**	Pagamentos para investigação de imagem (raios-x, tomografia computadorizada, ultrassom, etc.). Específicos para TB e outros.
	**TESTES LABORATORIAIS**	Pagamentos para todos os testes específicos para TB e outros
	**OUTROS PROCEDIMENTOS**	Pagamentos para biópsia, lavagem brônquica, etc..apenas para procedimento relacionados à TB.
	**TAXA DE MEDICAMENTO**	Qualquer medicamento prescrito antes de ser diagnosticado com tuberculose
	**PAGAMENTOS NÃO MÉDICOS**	
	**VIAGEM**	Pagamentos para deslocamento/viagens até o serviço de saúde, tanto para o paciente como para qualquer membro do agregado familiar/acompanhante.
	**ALIMENTAÇÃO**	Despesas com alimentação durante a viagem para cuidados de saúde, durante a visita ou hospitalização, tanto para pacientes como para quaisquer membros do agregado familiar/acompanhante.

	**ALOJAMENTO**	Pagamentos diretos relacionados com o aluguel de um quarto / cama durante as visitas de cuidados de saúde e quaisquer outros pagamentos não médicos relacionados com a visita de cuidados de saúde, tanto para o doente como para qualquer membro do agregado (hospedagem).
	**SUPLEMENTO NUTRICIONAL**	Valor pago por suplemento nutricional. Produtos que tem a finalidade de suplementar a dieta com nutrientes ou outras substâncias em situações específicas, como os suplementos vitamínicos e ou minerais.
	**REEMBOLSO DO SEGURO SAÚDE**	O montante reembolsado ao paciente através do seguro médico (privado ou previdenciário) até o momento, não inclui o reembolso futuro esperado.
	**PAGAMENTO DIRETO TOTAL (BRUTO)**	Pagamento direto aos prestadores de serviços de saúde por indivíduos no momento do uso do serviço, isto é, excluindo pagamento antecipado de serviços de saúde - por exemplo, na forma de impostos ou de prêmios ou contribuições de seguro específicos. Calculado como a soma dos custos médicos diretos (A) e diretos não médicos (B). Se o paciente não puder lembrar os detalhes dos custos acima, pedir o total de pagamentos da visita, hospitalização.
	**PAGAMENTO DIRETO (LIQUIDO)**	Pagamento direto médicos e não médicos menos o reembolso

					**PAGAMENTOS MÉDICOS** (Total por visita) (A)						
30.a)	30.b)	30.c) Tipo	30.d)	30.e)	30.f) Diárias	30.g)	30.h)	30.i) Testes	30.j) Outros	30.k) Medicamentos	Total de Pagamentos

Visita	Semanas antes do início do tratamento	de provedor	Tempo de viagem (horas)	Tempo gasto para visitar (horas)	(para internações somente) A1	Taxa de consulta A2	Radiografia e outras imagens A3	laboratoriais A4	procedimentos A5	A6	médicos. total ΣA1-6
1st											
2nd											
3rd											
4th											
5th											
6th											
7th											

					**PAGAMENTOS NÃO-MÉDICOS** (Total por visita) (B)					Total de pagamentos (A+B)	Reembolso de seguro de saúde (C)	30.r) Pagamento direto (Líquido) por visita (A+B-C)
30.a) Visita	30.b) Semanas antes do início do tratamento	30.c) Tipo de provedor	30.d) Tempo de viagem (horas)	30.e) Tempo gasto para visitar (horas)	30.l) Deslocamento / Viagens B1	30.m) Alimentação B2	30.n) Alojamento B3	30.o) Suplementos nutricionais B4	Total de pagamentos não-médicos (Total) ΣB1-4	30.q)Total dos pagamentos (ΣA1-6) + (ΣB1-4)	30.p) Reembolso de seguro de saúde	
1st												
2nd												
3rd												
4th												
5th												
6th												
7th												

PARTE III. CUSTO DURANTE O ATUAL TRATAMENTO DE TB/MDR-TB (DEVE SER PREENCHIDO PARA TODOS OS PACIENTES)

<table>
<tr><th>QUESTÃO</th><th>CATEGORIAS DE RESPOSTAS</th><th>ORIENTAÇÃO PARA O ENTREVISTADOR</th></tr>
<tr><td rowspan="3">31. Você foi hospitalizado, na fase atual do tratamento de TB e está hospitalizado por causa de TB?</td><td>1. Sim 31. b) _____dias.</td><td>Refere-se APENAS a hospitalização durante a fase de tratamento ATUAL: NÃO inclui internação antes de começar este tratamento.</td></tr>
<tr><td>2. Não</td><td>Internações anteriores ao diagnóstico de TB devem ser preenchidas em parte II deste formulário.</td></tr>
<tr><td colspan="2">CASO A RESPOSTA PARA A PERGUNTA 31 SEJA "NÃO", PULE PARA A PERGUNTA 33</td></tr>
<tr><td rowspan="6">32. Quanto de dinheiro e tempo você gastou para cada uma destas internações?</td><td colspan="2">VEJA A TABELA ABAIXO E PERGUNTE POR CADA ITEM:</td></tr>
<tr><td colspan="2">• Preencha uma linha por hospitalização;
• Para todos os que não se aplicam, marque / selecione NA
• Adicione mais linhas se mais hospitalizações ocorreram durante o tratamento da TB.

EXPLICAÇÃO DOS TÍTULOS DA TABELA:

Hospitalização: deve ser preenchida em ordem cronológica, primeira hospitalização = primeira linha.
Tipo de hospital: preencher o tipo de provedor de acordo com as categorias abaixo:
6. Unidade de Urgência e Emergência (Pronto Socorro - PS, Unidades de Pronto atendimento - PA)
7. Hospital Público
8. Serviço privado de saúde
9. Outro
Número de dias hospitalizados: deve ser preenchido em ordem cronológica</td></tr>
<tr><td colspan="2">PAGAMENTOS MÉDICOS</td></tr>
<tr><td>ENCARGOS DO DIA/DIÁRIA:</td><td>Apenas para hospitalizações pagas (particular).</td></tr>
<tr><td>TAXA DE CONSULTA:</td><td>Outros encargos, não cobertos pela taxa diária, incluindo pagamento direto ao pessoal de saúde.</td></tr>
</table>

	**RADIOGRAFIA E OUTRAS IMAGENS:**	Pagamentos para investigação de imagem (raios-x, tomografia computadorizada, ultrassom, etc...). Específicos para TB e outros.
	**TESTES LABORATORIAIS:**	Pagamentos para todos os testes, específicos para TB e outros, incluindo o custo de transporte de amostra, se pagas pelo paciente.
	**OUTROS PROCEDIMENTOS**	Pagamentos para biópsia, lavagem brônquica, etc., apenas para procedimentos relacionados à TB.
	**TAXAS DE MEDICAMENTOS**	Qualquer medicamento prescrito durante o tratamento para TB.
	**PAGAMENTOS NÃO MÉDICOS**	
	**VIAGEM**	Pagamentos para deslocamento/viagens até o hospital tanto para o paciente como para qualquer membro do agregado familiar/acompanhante.
	**ALIMENTAÇÃO**	Despesas com alimentação durante a viagem para cuidados de saúde, durante a hospitalização, tanto para pacientes como para quaisquer membros do agregado familiar/acompanhante.
	**ALOJAMENTO**	Pagamentos diretos relacionados com o aluguel de um quarto / cama durante a hospitalização e quaisquer outros pagamentos não médicos relacionados com a visita de cuidados de saúde, tanto para o paciente como para qualquer membro do agregado (hospedagem).
	**SUPLEMENTO NUTRICIONAL**	Valor pago por suplemento nutricional. São produtos que tem a finalidade de suplementar a dieta com nutrientes ou outras substâncias em situações específicas, como por exemplo suplementos vitamínicos e ou minerais, dietas específicas, etc.

	**OUTROS PAGAMENTOS**:	Pagamentos de roupas de cama, produtos de higiene pessoal, outros serviços.
	**REEMBOLSO DO SEGURO SAÚDE:**	O montante reembolsado ao paciente através do seguro médico (privado ou previdenciário) até o momento, não inclui o reembolso futuro esperado.
	**PAGAMENTO DIRETO TOTAL (BRUTO)**	Pagamento direto aos prestadores de serviços de saúde por indivíduos no momento do uso do serviço, isto é, excluindo pagamento antecipado de serviços de saúde - por exemplo, na forma de impostos ou de prêmios ou contribuições de seguro específicos. Calculado como a soma dos custos médicos diretos (A) e diretos não médicos (B). Se o paciente não puder lembrar os detalhes dos custos acima, pedir o total de pagamentos da visita, hospitalização.
	**PAGAMENTO DIRETO (LÍQUIDO)**	Pagamentos diretos médicos e não-médicos menos os reembolsos.

	**PAGAMENTOS MÉDICOS**<br>(Total por estadia)<br>(A)

Hospitalização	32.a) Tipo de hospital (ver lista)	32.b) Número de dias hospitalizado	32.c) Tempo de viagem (horas)	32.d) Encargas do dia (totais de estadia) A1	32.e) Taxa de consulta (total por estadia) A2	32.f) Radiografia e outras imagens (total por estadia) A3	32.g) Testes de laboratório (totais por estadia) A4	32.h) Outros procedimentos A5	32.i) Outros medicamentos (total de estadia) A6	Pagamentos médico (Total) ΣA1-6
1st										
2nd										
3rd										
4th										
5th										
6th										

				**PAGAMENTOS NÃO-MÉDICOS** (Total por estadia) (B)						Total dos pagamentos (A+B)	Reembolso de seguro de saúde (C)	32.q) Total de pagamentos (liquido) por estadia (A+B-C)
Hospitalização	32.a) Tipo de hospital (ver lista)	32.b) Número de dias hospitalizado	32.c) Tempo de viagem (horas)	32.j) Viagens (total por estadia) B1	32.k) Alimentos (total por estadia) B2	32.l) Alojamento (total por estadia) B3	32.m) Suplementos nutricionais B4	32.n) Outros pagamentos (total por estadia) B5	Total de pagamentos não-médicos (Total) ΣB1-5	32.p) Total dos pagamentos	32.o) Reembolso de seguro de saúde	
1st												
2nd												
3rd												
4th												
5th												
6th												

CUSTOS PARA TDO DURANTE AMBULATÓRIO		
**QUESTÃO**	**CATEGORIAS DE RESPOSTAS**	**ORIENTAÇÃO PARA O ENTREVISTADOR**
**33. Atualmente toma seus medicamentos sem supervisão ou apoio (auto administrado) ou tem um supervisor de tratamento (TDO)?**	1. Auto administrado<br>2. TDO<br>3. Auto administrado e TDO	TDO (Tratamento Diretamente Observado) é a supervisão da ingestão do medicamentos para Tuberculose por profissional de saúde ou familiar/pessoa amiga<br><br>**CASO "AUTO-ADMINISTRADO" PULE PARA A PERGUNTA 35.**
**34. Se TDO, quantos dias por semana?**	**1.** Sete dias por semana<br>**2.** Seis dias por semana<br>**3.** Cinco dias por semana<br>**4.** Quatro dias por semana<br>**5.** Três dias por semana<br>**6.** Dois dias por semana<br>**7.** Um dia na semana<br>**8.** Outro	Número de dias na semana em que o TDO é realizado.<br><br>Se outro, especificar.
**35. Se você está agora na fase de manutenção, tomou seus medicamentos na fase**		**Esta questão refere-se apenas a fase de manutenção do tratamento para Tuberculose.**

**intensiva sem supervisão ou apoio (auto administrado), ou você teve um supervisor de tratamento (TDO)?**	1. Auto administrado<br><br>2. TDO em fase intensiva ----------- dias por semana	Registrar o número de dias na semana em que o TDO foi realizado, conforme categorias da questão 34.<br><br>**CASO "AUTO ADMINISTRADO" PULAR PARA QUESTÃO 41.**
**36. Se TDO, quem é o provedor /supervisor do TDO?**	1. Profissional de Saúde da Unidade de saúde<br>2. Agente Comunitário de Saúde - ACS<br>3. Voluntário<br>4. Membro da família<br>5. Amigo<br>6. Outro	1. TDO realizado por uma pessoa especifica do serviço de saúde<br>2. TDO realizado por ACS<br>3. TDO realizado por pessoa voluntária no serviço de saúde ou não<br>4. TDO realizado por um membro da família<br>5. TDO realizado por um amigo<br>6. Outro – Especificar
**37. Se TDO, quanto tempo gastou na última visita, incluindo o tempo de viagem e tempo de espera (tempo de ida, volta e espera)?**	_______________ horas	Registrar o número total de horas para realizar TDO.

**38. Qual foi o custo de transporte (ida e volta) para a última visita de TDO, incluindo os custos de estacionamento, no total para você e qualquer membro do agregado que acompanha?**	R$ _____,______<br><br>Não lembra	Refere-se ao custo com deslocamento seja ele transporte público ou privado. Registrar o valor na moeda corrente.
**39. Havia alguma taxa paga ao provedor /supervisor do TDO?**	1. Sim / Se sim, registrar o valor R$ _____,____<br>2. Não	Refere-se ao custo com pessoal, recursos humanos, destinado ao TDO. Se, sim registrar o valor em moeda corrente.
**40. Quanto gastou com alimentos e bebidas para a última visita para TDO (na estrada, enquanto espera... almoço etc.), no total para você e qualquer membro de sua família ou acompanhante?**	R$ _____,______<br><br>Não lembra	Refere-se ao custo com o TDO, incluindo alimentação e deslocamento do paciente e acompanhante. Registrar o valor em moeda corrente.
**CUSTOS PARA RETIRADA DE MEDICAMENTOS DURANTE O ATENDIMENTO AMBULATORIAL**		

ATENÇÃO: Custos para retirada de medicamentos durante tratamento ambulatorial (devemos diferenciar a visita TDO e a visita de retirada de medicamentos para que não sejam confundidas uma com a outra – a retirada de medicamentos é uma visita muito mais curta do que a visita do TDO e não envolve necessariamente o mesmo tipo de cuidados de saúde e trabalhador.

**QUESTÃO**	**CATEGORIAS DE RESPOSTA**	**ORIENTAÇÃO PARA O ENTREVISTADOR**
41**. Você ou um membro do seu agregado familiar faz retirada de medicamentos para TB (para o tratamento auto administrado ou para trazer para o seu SUPERVISOR TDO)?**	1. Sim<br>2. Não	Isso não diz respeito às visitas ao TDO, que devem ser registradas nas perguntas 33-40, mas devem ser preenchidas se o paciente ou outro membro do domicílio pegam medicamentos para levar ao supervisor de TDO ou para tratamento auto administrado.<br>Se o paciente estiver em TDO e o paciente ou membro da família não estiver pegando medicamentos para levar ao supervisor do TDO, a resposta é não.<br>**CASO A RESPOSTA SEJA “NÃO”, PULE PARA A QUESTÃO 49.**
**42. Se sim. Quantas vezes que você ou um membro do agregado familiar faz a retirada de medicamentos para TB na fase atual de tratamento?**	1. Uma vez por semana<br>2. Duas vezes por mês<br>3. Uma vez por mês<br>4. Uma vez a cada dois meses<br>5. Outro_______________	Periodicidade em que o paciente ou membro da família faz a retirada de medicamentos para o tratamento de TB. Outro, especificar.

**43. Onde você ou seu familiar/acompanhante retira seus medicamentos para TB?**	1. Unidade de Saúde da família<br>2. Unidade Básica de saúde<br>3. Unidade de Referência para tuberculose<br>4. Unidade de Cuidados de Urgência e Emergência<br>5. Hospital público<br>6. Hospital privado<br>7. Serviço de atenção farmacêutica<br>8. Outro	***OBSERVAR*** a diferenciação em serviços de saúde da Atenção Primária (USF, UBS, PCT,) e Serviços Especializados (PAs, Ponto Socorro, Hospital Público).<br><br>Se o paciente tem visitado lugares diferentes, assinale o que for mais recente.
**44. Havia alguma taxa paga para retirada de medicamentos?**	1. Sim<br>Se sim, R$ _____,______<br><br>2. Não<br><br>Não lembra	Registrar o valor na moeda corrente
**45. Que custos de alojamento você e/ou qualquer membro do agregado familiar ou acompanhante teve quando foi retirar medicamentos na última vez?**	R$ _____,______<br><br>Não lembra	Registrar o valor na moeda corrente

**46. Quanto tempo a última visita de retirada de medicamentos levou, incluindo o tempo de viagem e tempo de espera (tempo de ida, volta e espera total)?**	_____________ horas	Registrar o número total de horas para retirada de medicamentos.
**47. Qual foi o custo do transporte (ida e volta) da última vez que você retirou medicamentos, incluindo os custos de estacionamento, no total para você e qualquer membro do agregado familiar que o acompanha?**	R$ _____,______<br><br>Não lembra	Registrar o valor na moeda corrente
**48. Quanto gastou com alimentos e bebidas da última vez que você retirou medicamentos (na estrada, enquanto esperava... almoço etc.), no total para você e qualquer membro do agregado familiar ou acompanhante.**	R$ _____,______<br><br>Não lembra	Registrar o valor na moeda corrente
**CUSTO DURANTE AS VISITAS PARA ACOMPANHAMENTO MÉDICO AMBULATORIAL (CONSULTA AO MÉDICO OU ENFERMEIRO, EXAMES)**		

QUESTÃO	CATEGORIAS DE RESPOSTA	ORIENTAÇÃO PARA O ENTREVISTADOR
**49. Quantas consultas de acompanhamento relacionadas à FASE ATUAL de tratamento para TB você teve até agora?**	______consultas	Registrar o número de consultas (Ex: 1,2, 3 etc..) Isso se refere a check-up clínico, acompanhamento e visitas adicionais devido a efeitos colaterais ou outros problemas relacionados à tuberculose. Não inclui visitas ao TDO ou visitas para pegar medicamentos. Para pacientes em fase de continuação, quantas visitas desde o início da fase intensiva. (para consultar o médico ou enfermeira, fazer exames de acompanhamento, etc.)
**50. Quanto tempo levou para o deslocamento (viagem - ida e volta) até o estabelecimento de saúde em visita de acompanhamento?**	_________ horas	Registrar o número total de horas. Ida e volta.
**51. Quanto tempo que você permaneceu no serviço de saúde (esperando) desde o momento em que você chegou em visita de acompanhamento?**	__________ horas	Registrar o número total de horas para assistência do pessoal do serviço de saúde ao paciente
**52. Qual foi o custo de transporte (ida e volta) para a última visita de acompanhamento, incluindo estacionamento, no total para você e qualquer membro do agregado familiar ou**	R$ _____,______<br><br>Não lembra	Registrar o valor na moeda corrente<br><br>Custo relacionado com a última visita.<br><br>Se a entrevista ocorrer no final de uma visita, então use os custos para a presente visita (ida e volta)

**acompanhante?**		
**53. Qual o custo de acomodação que teve para a última visita de acompanhamento, no total, para você e qualquer membro do agregado familiar ou acompanhante?**	R$ _____,______<br><br>Não lembra	Registrar o valor na moeda corrente<br><br>Custo relacionado com a última visita.<br><br>Se a entrevista ocorrer no final de uma visita então use os custos para a presente visita
**54. Quanto você pagou durante sua última visita (consulta) de acompanhamento?**	R$ _____,______<br><br>Não lembra	Registrar o valor na moeda corrente. Custo relacionado com a última visita. Se a entrevista ocorrer no final de uma visita então use os custos para a presente visita. Acompanhamento para qualquer profissional de saúde.
**55. Quanto você pagou durante sua última visita de acompanhamento para radiografia e outras imagens?**	R$ _____,______<br><br>Não lembra	Registrar o valor na moeda corrente Radiografia e outras imagens: pagamentos para investigação de imagem (raios-x, tomografia computadorizada, ultrassom). Específicos para TB e outros
**56. Quanto que você pagou durante sua última visita de acompanhamento para testes de TB e outros?**	R$ _____,______<br><br>Não lembra	Registrar o valor na moeda corrente Custo relacionado com a última visita. Se a entrevista ocorrer no final de uma visita então use os custos para a presente visita.

**57. Quanto você pagou na sua última visita de acompanhamento para medicamentos para TB, incluindo prescrições de medicamentos comprados fora do serviço de saúde?**	R$ _____,______<br><br>Não lembra	Registrar o valor na moeda corrente Custo relacionado com a última visita. Se a entrevista ocorrer no final de uma visita então use os custos para a presente visita.
**58. Quanto que você pagou durante sua última visita de acompanhamento para outros procedimentos?**	R$ _____,______<br><br>Não lembra	Registrar o valor na moeda corrente. Custo relacionado com a última visita.<br>Se a entrevista ocorrer no final de uma visita então use os custos para a presente visita.
**59. Quanto que você pagou durante sua última visita de acompanhamento para outros medicamentos, incluindo os suplementos nutricionais?**	R$ _____,______<br><br>Não lembra	Registrar o valor na moeda corrente Custo relacionado com a última visita.<br>Se a entrevista ocorrer no final de uma visita então use os custos para a presente visita. Outros medicamentos que não os específicos para o tratamento da TB
**60. Que outras gastos não constantes as perguntas anteriores você pagou durante sua última visita de acompanhamento?**	R$ _____,______<br><br>Não lembra	Registrar o valor na moeda corrente Custo relacionado com a última visita.<br>Se a entrevista ocorrer no final de uma visita então use os custos para a presente visita.
**CUSTOS PARA SUPLEMENTOS NUTRICIONAIS / ALIMENTARES**		

**61. Você compra/comprou quaisquer suplementos nutricionais fora de sua dieta regular devido a doença TB, por exemplo, vitaminas recomendadas pela equipe de cuidados de saúde?**	1. Sim<br>2. Não	**CASO A RESPOSTA SEJA “NÃO”, PULE PARA A QUESTÃO 63.**
**62. Se sim, quanto gastou com estes suplementos nutricionais na semana passada e no último mês aproximadamente?**	62. a) R$ _____,______ semana ou Não lembra<br>62. b) R$ _____,______ mês ou Não lembra	Registrar o valor na moeda corrente no último mês
**63. Você compra/comprou qualquer alimento adicional fora de sua dieta regular devido a doença TB, por exemplo, carne, bebidas energéticas ou frutas, conforme recomendado pela equipe de cuidados de saúde na semana passada aproximadamente?**	1. Sim<br>2. Não	**CASO A RESPOSTA SEJA ”NÃO”, PULE PARA A PERGUNTA 65**

**64. Se sim, quanto gastou com essa comida adicional na semana passada aproximadamente?**	R$ _____,______<br><br>Não lembra	Registrar o valor na moeda corrente

PERDA DE TEMPO PARA ACOMPANHANTES

Perda de tempo relacionada à educação não deve ser preenchida se o paciente é inferior a 18 anos para crianças. Todas as questões relativas a custos, tempo gasto, renda, e a perda de rendimento nas secções II e o custo de preocupação III para o guardião ou responsável.

QUESTÃO	CATEGORIAS DE RESPOSTAS	ORIENTAÇÃO PARA O ENTREVISTADOR
**65. Alguém da sua família o acompanha:**	1. Sim	

**65. a) Visita TDO**	2. Não	**ASSINALAR UMA OPÇÃO (SIM/NÃO) PARA CADA ITEM DA QUESTÃO.**<br><br>**CASO A RESPOSTA SEJA "NÃO", VÁ PARA A QUESTÃO 67.**
**65. b) Visita para buscar medicamentos (ou buscar os medicamentos para você)**	1. Sim<br>2. Não	
**65. c) Visita de consulta médica**	1. Sim<br>2. Não	
**65. d) Hospitalização**	1. Sim<br>2. Não	
**66. Se sim (para qualquer alternativa anterior), essa pessoa perdeu uma renda durante esse tempo?**	1. Sim<br>2. Não	Perder uma renda seria perder valor por tempo de trabalho perdido. Valor de horas perdidas de trabalho vezes o valor em reais da hora de trabalho. Caso haja várias respostas na questão 65, perguntar sobre a mais recente visita que um membro do agregado familiar o acompanhou.
**REGIME DE SEGURO DE SAÚDE**		
**QUESTÃO**	**CATEGORIAS DE RESPOSTAS**	**ORIENTAÇÃO PARA O ENTREVISTADOR**

**67. Tem qualquer um dos seguintes tipos de seguro de saúde/plano de saúde?**	1. Nenhum<br>2. Regime de reembolso<br>3. Coparticipação ou cobertura parcial<br>4. Cobertura total<br>5. Outro ___________	1. Não há plano de saúde ou qualquer regime de reembolso<br>2. Tem plano de saúde com regime de reembolso (ressarcimento)<br>3. Tem plano de saúde com coparticipação ou parcial, o usuário paga além do valor mensal, uma taxa a cada vez que passa por consulta ou faz exames.<br>4. Tem plano de saúde com cobertura total, não paga taxa adicional por consulta ou exames, além do valor mensal.<br>5. Se outros, especificar.
**POSIÇÃO SOCIAL**		
**QUESTÃO**	**CATEGORIAS DE RESPOSTAS**	**OREINTAÇAO PARA O ENTREVISTADOR**<br>**(se o paciente é menor de 18 anos de idade, estas perguntas dizem respeito a guardião ou responsável.**
**68. Qual é seu nível de escolaridade (do paciente)?**	1. Analfabeto / Fundamental Incompleto<br>2. Fundamental I completo / Fundamental II incompleto<br>3. Fundamental II completo / Médio incompleto<br>4. Médio Completo / Incompleto Superior<br>5. Completo Superior	Se o paciente é menor de 18 anos, esta pergunta é para o responsável.<br><br>ABEP - Associação Brasileira de Empresas de Pesquisa – 2018 – www.abep.org – abep@abep.org

**69. Qual é a sua ocupação principal?**	1. Estudante<br>2. Trabalhador<br>3. Agricultor<br>4. Aposentado<br>5. Doméstico<br>6. Desempregado<br>7. Outros	Se o paciente é menor de 18 anos, esta pergunta é para o responsável.<br>Se outros, especificar.
**70. Qual foi o seu emprego principal ou trabalho formal, ou outra atividade principal antes de você contrair TB?**	1. Nenhum<br>2. Trabalho doméstico<br>3. Militares de exército, marinha, aeronáutica, polícia militar ou fogo militar<br>4. Emprego no setor privado<br>5. Emprego no setor público<br>6. Empregador<br>7. Trabalha por conta própria (autônomo)<br>8. Trabalhador desempregado recebe ajuda do membro do agregado familiar<br>9. Estudante<br>10. Outros	Se o paciente é menor de 18 anos, esta pergunta é para o guardião ou responsável.<br>Isto se refere ao tempo antes de desenvolver sintomas de TB.<br>Se outros, especificar.<br>**Retirado do POF 5 – Questionário de aquisição coletiva (2017-2018) - sequência de perguntas número 53. B.2.**

**71. Qual é o seu emprego principal? Ou trabalho formal, ou outra atividade principal agora?**	1. Nenhum<br>2. Trabalho doméstico<br>3. Militares de exército, marinha, aeronáutica, polícia militar ou fogo militar<br>4.Emprego no setor privado<br>5. Emprego no setor público<br>6. Empregador<br>7. Trabalha por conta própria (autônomo)<br>8. Trabalhador desempregado recebe ajuda do membro do agregado familiar<br>9. Estudante<br>10. Outros	Se o paciente é menor de 18 anos, essa pergunta é para o guardião ou responsável.<br>Isso se refere ao tempo atual<br>Se outros, especificar.<br><br>**Retirado do POF 5 – Questionário de aquisição coletiva (2017-2018) - sequência de perguntas número 53. B.2.**
**As perguntas a seguir são para medir a capacidade do agregado familiar de pagar pela saúde usando três métodos:**<br><br>**1) Despesa de consumo (medida 1) // 2) Ativos domésticos e nível de educação (medida 2) // 3) e renda autodeclarada (medida 3)**		
**72. Sua casa tem?**	**Se sim, quantos**	**Assinalar uma opção (Sim/Não) para cada item da questão. Se sim, escrever quantos em números. Itens retirados do CCEB - pontuações**

**72. a) Banheiro**	1. Sim, ______ 2. Não	**A. Banheiro** - O que define o banheiro é a existência de vaso sanitário. Considerar todos os banheiros e lavabos com vaso sanitário, incluindo os de empregada, os localizados fora de casa e os da(s) suíte(s). Para ser considerado, o banheiro tem que ser privativo do domicílio. Banheiros coletivos (que servem a mais de uma habitação) não devem ser considerados.
**72.b) Veículo com quatro rodas**	1. Sim, ______ 2. Não	**B. Automóvel** - Não considerar táxis, vans ou pick-ups usados para fretes, ou qualquer veículo usado para atividades profissionais. Veículos de uso misto (pessoal e profissional) não devem ser considerados
**72. c) Computador**	1. Sim, ______ 2. Não	**C. Microcomputador** - Considerar os computadores de mesa, laptops, notebooks e netbooks. Não considerar: calculadoras, agendas eletrônicas, tablets, palms, smartphones e outros aparelhos;
**72. d) Lava-louças**	1. Sim, ______ 2. Não	D.**Lava-Louça** - Considere a máquina com função de lavar as louças;
**72. e) Geladeira**	1. Sim, ______ 2. Não	E. **Geladeira;**
**72.f) Congelador**	1. Sim, ______ 2. Não	**F. FREEZER; GELADEIRA E FREEZER**- No quadro de pontuação há duas linhas independentes para assinalar a posse de geladeira e freezer respectivamente. independente: Havendo uma geladeira no domicílio, serão atribuídos os pontos (SIM) correspondentes a posse de geladeira; Se a geladeira tiver um freezer incorporado –2ª porta– ou houver no domicílio um freezer independente serão atribuídos os pontos (SIM) correspondentes ao freezer;

**72.g) Máquina de lavar**	1. Sim, ______ 2. Não	**G. LAVA-ROUPA -**Considerar máquina de lavar roupa, somente as máquinas automáticas e/ou semiautomática. O tanquinho NÃO deve ser considerado;
**72.h) DVD**	1. Sim, ______ 2. Não	**H. DVD**- Considere como leitor de DVD (Disco Digital de Vídeo ou Disco Digital Versátil) o acessório doméstico capaz de reproduzir mídias no formato DVD ou outros formatos mais modernos, incluindo videogames, computadores, notebooks. Inclua os aparelhos portáteis e os acoplados em microcomputadores. Não considere DVD de automóvel;
**72.i) Microondas**	1. Sim, ______ 2. Não	**I. MICRO-ONDAS**- Considerar forno micro-ondas e aparelho com dupla função (de micro-ondas e forno elétrico);
**72.j) Moto**	1. Sim, ______ 2. Não	**J. MOTOCICLETA**- Não considerar motocicletas usadas exclusivamente para atividades profissionais. Motocicletas apenas para uso pessoal e de uso misto (pessoal e profissional) devem ser consideradas;
**72.k) Máquina de secar roupa**	1. Sim, ______ 2. Não	**K. SECADORA DE ROUPAS**- Considerar a máquina de secar roupa. Existem máquinas que fazem duas funções, lavar e secar. Nesses casos, devemos considerar esse equipamento como uma máquina de lavar e como uma secadora.

**73. O seu agregado familiar emprega trabalhadores domésticos?**	1. Sim<br>2. Não	**EMPREGADOS DOMÉSTICOS**– considerar apenas os empregados mensalistas, isto é, aqueles que trabalham pelo menos cinco dias por semana, durmam ou não no emprego. Não esqueça de incluir babás, motoristas, cozinheiras, copeiras, arrumadeiras, considerando sempre os mensalistas.<br>**NOTE BEM:** o termo empregado mensalista se refere aos empregados que trabalham no domicílio de forma permanente e/ou continua, pelo menos cinco dias por semana, e não ao regime de pagamento do salário.
**74. O seu agregado familiar tem acesso à água canalizada?**	1. Sim<br>2. Não	Rede de abastecimento de água potável
**75. Sua casa está em uma rua pavimentada?**	1. Sim<br>2. Não	Rua calçada, asfaltada

RENDIMENTO AUTODECLARADO ANTES DE CONTRAIR TB

Essas perguntas permitirão estimar a capacidade de pagamento da família – Medida 3.

Em um país como o Brasil com tamanho substancial de trabalho informal, as respostas a essas perguntas serão examinadas criticamente e comparadas com a renda estimada com base nas despesas de consumo (ou seja, “Capacidade de pagamento - medida 1”) e propriedade de bens com nível de escolaridade

**76. Você foi à pessoa que obteve a maior renda em sua casa antes de contrair TB?**	1. Sim<br>2. Não, outra pessoa no agregado familiar tinha um rendimento mais elevado.<br>3. Não, a renda era semelhante a outros membros da família	Se o paciente é menor de 18 anos, essa pergunta é para o responsável.
**77. Quantas horas por semana você estava trabalhando antes de contrair TB?**	__________ horas	Se o paciente é menor de 18 anos, essa pergunta é para o guardião ou responsável. Isso se refere ao tempo antes do desenvolvimento dos sintomas de TB.
**78. Se você estava trabalhando, quanto você <u>recebia</u> com atividades relacionadas ao trabalho, por mês antes de você contrair TB (rendimento líquido)?**	R$ _____,______<br>Não lembra	Para trabalhadores sazonais que experimentam salários flutuantes, tente determinar uma renda mensal média para essa questão. Rendimento líquido é o salário bruto mais os descontos previstos em legislação trabalhistas (impostos).
**79. Quanto você estima o rendimento de sua residência por mês relacionado ao trabalho, antes de contrair tuberculose? (A renda líquida de todos os membros da família deve ser contada)**	R$ _____,______<br>Não lembra	Refere-se a todas as pessoas da casa. Para trabalhadores sazonais que experimentam salários flutuantes, tente determinar uma renda mensal média para essa questão. Rendimento líquido, é o salário bruto mais os descontos previstos em legislação trabalhistas (impostos). Não relacionado a renda de pensão, aluguel, benefício social e outros rendimentos

MUDANÇAS DE RENDA E CONSEQUÊNCIAS SOCIAIS

Categorias de respostas e Orientação para o entrevistador (circule o mais adequado ou preencha a resposta na linha de resposta). Caso o paciente tem menos de 18 anos de idade, essas questões dizem respeito ao guardião ou responsável.

**80. Durante a fase intensiva, você teve que mudar de emprego ou profissão ou parou de trabalhar?**	1. Sim<br>2. Não	A fase intensiva, ou fase inicial do tratamento, tem o objetivo de reduzir rapidamente a população bacilar e a eliminação de bacilos com resistência natural a algum medicamento, e como consequência a redução da contagiosidade.
**81. Durante a fase de continuação, você teve que mudar de emprego ou profissão ou parou de trabalhar?**	1. Sim<br>2. Não	A fase de manutenção, após a fase inicial ou intensiva. Tem como objetivo de eliminar os bacilos latentes ou persistentes e a redução da possibilidade de recidiva da doença.
**82. Quanto você estima o rendimento de sua residência por mês relacionado ao trabalho, no momento do seu diagnóstico (líquido)?**	R$ _____,______ou Não lembra	Registrar o valor na moeda corrente, no decorrer de um mês.<br><br>Refere-se a todas as pessoas da casa. Para trabalhadores sazonais que experimentam salários flutuantes, tente determinar uma renda mensal média para essa questão.
**83. Se você estava trabalhando, quanto você recebe com atividades relacionadas ao trabalho, por mês agora (líquido)?**	R$ _____,______ou Não lembra	Se o paciente tem menos de 18 anos, essa pergunta é para o guardião ou responsável.<br><br>Lucro líquido refere-se ao valor recebido com os descontos devidos ao fisco.

**84. Quanto você estima o rendimento de atividades relacionadas ao trabalho de sua casa por mês agora (líquido)?**	R$ _____,______ ou Não lembra	Refere-se a todas as pessoas no domicílio. Registrar o valor na moeda corrente. Para trabalhadores sazonais que experimentam salários flutuantes, tente determinar uma renda mensal média.
**85. Quantas horas por semana você está trabalhando agora?**	_____ (horas)	Registrar o número total de horas trabalhadas por semana. Se o paciente é menor de 18 anos, esta questão é para o guardião ou responsável.<br>Se o paciente estiver desempregado, registrar como zero horas.
**86. Aproximadamente quantos dias úteis de renda você perdeu devido à sua doença de TB em geral?**	________(dias)	Registrar dias úteis perdidos pela doença, incluindo todos os dias antes e depois da perda de emprego.<br>Consideramos dias úteis todos os dias da semana, exceto sábados, domingos e feriados. Ex: considerar para um mês, sem feriados, 24 dias úteis (6 dias úteis x 4 semanas).
**87. Teve que pagar pelo trabalho perdido?**	**87. a) Antes do diagnóstico**<br>1. Sim<br>2. Não<br>**87. b) Agora**<br>1. Sim<br>2. Não	Registrar se houve perdas financeiras referente ao trabalho, antes do diagnóstico de TB e neste momento.
**88. Você ou sua família recebem algum pagamento de assistência social?**	1. Sim<br>2. Não	Se o paciente tem menos de 18 anos, essa pergunta é para o guardião ou responsável.

**Se sim. Que tipo e qual a quantia durante o último mês?**	**88.2**. Auxílio doença R$ ___,____.	**2. Auxílio doença:** benefício por invalidez devido ao segurado do Instituto Nacional do Seguro Social (INSS) que, em exame médico, demonstra estar temporariamente impossibilitado de trabalhar em decorrência de doença ou acidente.
	**88.3.** Benefício básico da bolsa família R$ ___,____.	**3. Benefício básico do bolsa família:** concedido a famílias vivendo em extrema pobreza (com renda mensal de até R$ 89,00 por pessoa). O auxílio é de R$ 89,00 mensais.
	88.**4**. Benefício variável R$ ___,____ ____.	**4. Benefício variável:** ou famílias em situação de pobreza ou extrema pobreza, gestantes, nutrizes (mães que amamentam), crianças e adolescentes de 0 a 15 anos de idade. O valor de cada benefício é de R$ 41,00 e cada família pode acumular até 5 benefícios por mês, chegando a R$ 205,00.
	**88.5**. Benefício variável de 0 a 15 anos do bolsa família R$ ___,____.	**5. Benefício variável de 0 a 15 anos do bolsa família**: destinado a famílias que tenham em sua composição crianças e adolescentes de 0 a 15 anos de idade. O valor do benefício é de R$ 41,00.
	**88.6**. Benefício variável à gestante do bolsa família R$ ___,____.	**6. Benefício variável à gestante do bolsa família:** Destinado a famílias com mulheres grávidas. Até nove parcelas consecutivas podem ser pagas a partir da data do início do pagamento do benefício, desde que a gestação tenha sido identificada até o nono mês. O valor do benefício é de R$ 41,00.

	**88.7**. Benefício variável à nutriz do bolsa família R$ ___,____.	**7. Benefício variável à nutriz do bolsa família**: Destinado a famílias com mulheres grávidas. Até nove parcelas consecutivas podem ser pagas a partir da data do início do pagamento do benefício, desde que a gestação tenha sido identificada até o nono mês. O valor do benefício é de R$ 41,00
	**88.8**. Benefício variável ao jovem do bolsa família R$ ___,____.	**8. Benefício variável jovem do bolsa família**: Destinado a famílias em situação de pobreza ou extrema pobreza e tendo na sua composição adolescentes entre os 16 e os 17 anos. O valor do benefício é de R$ 48,00 por mês e cada família pode acumular até dois benefícios, ou R$ 96,00.
	**88.9**. Benefício para superação da extrema pobreza do bolsa família R$ ___,____ .	**9. Benefício para superação da extrema pobreza do bolsa família:** Destinado a famílias que vivem em extrema pobreza. Cada família pode receber um benefício por mês. O valor do benefício varia de acordo com o cálculo feito da renda por pessoa da família e do benefício já recebido no Programa Bolsa Família.
	**88.10**. Benefício de Prestação Continuada BPC ____ por mês.	**10. Benefício de Prestação Continuada BPC**: garantia de um salário mínimo mensal para a pessoa com deficiência e para os idosos com 65 anos ou mais de baixa renda.
	**88.11**. Reembolso de seguro de saúde privado R$ ___,____.	**11. Reembolso de seguro de saúde privado**: Valores devolvidos de seguro de saúde privado.
	**88.12**. Licença médica remunerada R$ ___,____ .	**12. Licença médica remunerada**: licença quando doente, o que não lhe permite cumprir as funções do cargo, sendo possível concedê-lo sem prejuízo da sua remuneração.

	**88.13**. Benefício por invalidez R$ ___,____ .	**13. Benefício por invalidez**: benefício devido ao trabalhador permanentemente incapaz de realizar qualquer atividade de trabalho e que também não pode ser reabilitado em outra profissão, de acordo com a avaliação da perícia médica do INSS.
	**88.14**. Outro R$ ___,____ ____.	Retirado do sistema de benefício governamental MS
**89. Você atualmente recebe vales ou bens para lidar com a doença da tuberculose?**<br><br>**89. b) Quais**	1. Sim<br>A. Vale transporte<br>B. Cesta de alimentos<br>C. Outros<br><br>2. Não	Se o paciente é menor de 18 anos, essa pergunta é para o guardião ou responsável.<br><br>Mais de uma categoria permitida.<br><br>**CASO A RESPOSTA SEJA “NÃO”, PULE PARA A QUESTÃO 91.**
**90. De quem você recebe o vale / bens**	1. Governo<br>2. ONGs<br>3. Empregador<br>4. Doação privada<br>5. Outro	Se o paciente tem menos de 18 anos, essa pergunta é para o guardião ou responsável.<br><br>É permitido o preenchimento de mais de uma categoria. Selecione, tudo o que é mencionado.<br><br>Se outros, especificar.

**91. Quantos adultos e crianças dormem regularmente em sua casa? (incluindo paciente, se variável, usar no momento do diagnóstico)**	93.a. ____ adultos<br>93.b. _____ crianças	Registrar o número de pessoas, crianças e adultos que dormem no mesmo domicílio do paciente em tratamento de tuberculose.
**92. Quantos cômodos existem na casa, excluindo o banheiro?**	____	Registrar o número de cômodos da casa, excluindo o banheiro
**93. A doença TUBERCULOSE afetou sua vida social ou privada de alguma forma?**	1. Não<br>2. Com dificuldade em aquisição de alimentos<br>3. Divórcio ou separado do cônjuge / parceiro<br>4. Perda de emprego<br>5. Teve que parar de estudar<br>6. Exclusão social<br>7. Outro __________________	É permitido o preenchimento de mais de uma categoria. **Selecione** tudo o que é mencionado.<br><br>Se outros, especificar.
**ENFRENTAMENTO**		
**QUESTÃO**	**CATEGORIAS DE RESPOSTAS**	**ORIENTAÇÃO PARA O ENTREVISTADOR**
Se o paciente tiver menos de 18 anos, estas perguntas são para o guardião (responsável).		

**94. Você pegou emprestado ou recebeu algum dinheiro para cobrir os custos incorridos desde que iniciou o tratamento para TB?**	1. Sim<br>2. Não	**CASO A RESPOSTA SEJA "NÃO", VÁ PARA A QUESTÃO 98.**
**95. Se sim, quanto você recebeu (no total)?**	R$ _____,______ ou Não lembra.	Registrar o valor na moeda corrente.
**96. De quem você pediu / recebeu?**	1. Família / parente próximo<br>2. Parente distante<br>3. Vizinhos / amigos<br>4. Organização diferente de financiamento bancário<br>5. Loja de penhores<br>6. Empregador<br>7. Banco<br>8. "Credor não oficial" (agiota)<br>9. Outra	É permitido o preenchimento de mais de uma categoria. **Selecione** tudo o que é mencionado.<br><br>Se outros, especificar.
**97. Você teve/terá que pagar a quantia de volta?**	1. Sim<br>2. Não	Registrar se o paciente ou responsável teve que devolver o valor pego por empréstimo ou ter recebido durante tratamento de tuberculose
**98. Você já vendeu alguma propriedade para financiar o custo incorrido durante o tratamento da TB?**	1. Sim<br>2. Não	**CASO A RESPOSTA SEJA "NÃO", PULE PARA A QUESTÃO 101**

**99. Se sim, o que você vendeu?**	1. Terra<br>2. Pecuária<br>3. Transporte / veículo<br>4. Item doméstico<br>5. Produção Agrícola<br>6. Ouro / jóias<br>7. Outros	É permitido o preenchimento de mais de uma categoria. Selecione tudo o que é mencionado.<br><br>Se outros, especificar.
**100. Quanto dinheiro você recebeu com a venda de todos os itens de sua propriedade (no total)?**	R$ _____,______ou Não lembra .	Registrar o valor na moeda corrente.
**101. O impacto financeiro na sua casa desde que você teve sintomas de tuberculose foi que sua família se tornou:**	1. Muito mais rico<br>2. Mais rico<br>3. Inalterado<br>4. Mais pobre<br>5. Muito mais pobre	
**DESPESA DE CONSUMO DAS FAMÍLIAS (MEDIDA 1)**		

Essas perguntas permitem estimar a capacidade de pagamento da família. Perguntas foram extraídas de “Pesquisa de Orçamentos Familiares Simplificada Teste-piloto 2009”		
**DESPESAS SEMANAIS DE CONSUMO - 7 DIAS**		
**102. Por favor, dê a sua despesa SEMANAL em alguns itens como alimentos e bebidas.**	102. a) Alimentos R$ _____,______ ou Não lembra<br><br>102. b) Bebidas R$ _____,______ ou Não lembra	**ALIMENTOS:** arroz, feijão, carne de boi, carne de porco, peixe, aves, pão, macarrão, óleo, biscoito, café, açúcar, sal e condimentos, queijo, laranja, banana, maçã, verduras, doces e outros.<br><br>**BEBIDAS**: leite, suco de fruta, refrigerante, vinho, chá, aguardente, cerveja, água mineral, outros.<br><br>Registrar o valor na moeda corrente.<br><br>Total gasto com alimento e bebidas em **7 dias**;<br>Total gasto com outros produtos durante **7 dias.**<br>**POF 3 - 2017-2018**
**AQUISIÇÃO MÉDIA MENSAL FAMILIAR NO PERÍODO DE REFERÊNCIA - 12 MESES**		
Só preencha o valor médio anual quando não for possível estimar o Valor Médio Mensal<br><br>FORMA DE AQUISIÇÃO: 1 - monetária à vista; 2 - monetária a prazo; 3 - doação; 4 - produção própria; 5 - outras		
**HABITAÇÃO - DOMICÍLIO PRINCIPAL**		

**103. Energia elétrica**	103. a) Valor médio **MENSAL**: R$ __________ ou não lembra	103. b) Forma de aquisição:
	103. c) Valor médio **ANUAL:** R$ __________ ou não lembra	103. d) Forma de aquisição:
**104. Água e esgoto**	104. a) Valor médio **MENSAL**: R$ __________ ou não lembra	104. b) Forma de aquisição:
	104. c) Valor médio **ANUAL**: R$ __________ ou não lembra	104. d) Forma de aquisição:
**105. Gás (encanado ou de botijão) e outros combustíveis domésticos**	105. a) Valor médio **MENSAL:** R$ __________ ou não lembra	105. b) Forma de aquisição:
	105. c) Valor médio **ANUAL:** R$ __________ ou não lembra	105. d) Forma de aquisição:
**106. Aluguel e condomínio**	106. a) Valor médio **MENSAL**: R$ __________ ou não lembra	106. b) Forma de aquisição:
	106. c) Valor médio **ANUAL**: R$ __________ ou não lembra	106. d) Forma de aquisição:
**107. Prestação do imóvel**	107.a) Valor médio **MENSAL:** R$ __________ ou não lembra	107.b) Forma de aquisição:

	107.c) Valor médio **ANUAL:** R$ __________ ou não lembra	107.d) Forma de aquisição:
**108. IPTU**	108.a) Valor médio **MENSAL:** R$ __________ ou não lembra	108.b) Forma de aquisição:
	108.c) Valor médio **ANUAL:** R$ __________ ou não lembra	108.d) Forma de aquisição:
**109. Conservação, manutenção e pequenos reparos do domicílio principal (material mão-de-obra e ferramentas)**	109.a) Valor médio **MENSAL:** R$ __________ ou não lembra	109.b) Forma de aquisição:
	109.c) Valor médio **ANUAL:** R$ __________ ou não lembra	109.d) Forma de aquisição:
**110. Construção e reforma do domicílio principal**	110.a) Valor médio **MENSAL:** R$ __________ ou não lembra	110.b) Forma de aquisição:
	110.c) Valor médio **ANUAL:** R$ __________ ou não lembra	110.d) Forma de aquisição:
**111. Outros (conserto de móveis e equipamentos, outros impostos e taxas, mudança, seguro do imóvel, etc.)**	111.a) Valor médio **MENSAL:** R$ __________ ou não lembra	111.b) Forma de aquisição:
	111.c) Valor médio **ANUAL:** R$ __________ ou não lembra	111.d) Forma de aquisição:
**HABITAÇÃO - OUTROS IMÓVEIS**		
**112. Serviços e taxas (energia, água, aluguel, etc.) de outros**	112.a) Valor médio **MENSAL:** R$ __________ ou não lembra	112.b) Forma de aquisição:
	112.c) Valor médio **ANUAL:** R$ __________ ou não	112.d) Forma de aquisição:

**imóveis**	lembra	
**113. Conservação, manutenção e pequenos reparos de outros imóveis (material, mão de obra e ferramentas)**	113.a) Valor médio **MENSAL:** R$ __________ ou não lembra	113.b) Forma de aquisição:
	113.c) Valor médio **ANUAL:** R$ __________ ou não lembra	113.d) Forma de aquisição:
**114. Construção e reforma de outros imóveis ou de jazigo**	114.a) Valor médio **MENSAL:** R$ __________ ou não lembra	115.b) Forma de aquisição:
	114.c) Valor médio **ANUAL:** R$ __________ ou não lembra	115.d) Forma de aquisição:
**MÓVEIS, ELETRODOMÉSTICOS E OUTROS ITENS**		
**115. Móveis (cama, mesa, armário, etc.)**	115.a) Valor médio **MENSAL:** R$ __________ ou não lembra	115.b) Forma de aquisição:
	115.c) Valor médio **ANUAL:** R$ __________ ou não lembra	115.d) Forma de aquisição:
**116. Utensílios e enfeites**	116.a) Valor médio **MENSAL:** R$ __________ ou não lembra	116.b) Forma de aquisição:
